# Evidence‐Based Lessons From Two Decades of Implementation Research on Complementary Feeding Programmes

**DOI:** 10.1111/mcn.13811

**Published:** 2025-02-28

**Authors:** Tina G. Sanghvi, Sandra Remancus, Edward A. Frongillo, Rafael Perez‐Escamilla, Chessa Lutter, Pooja Pandey Rana, Victor Ogbodo, Tuan Nguyen, Roger Mathisen

**Affiliations:** ^1^ Alive and Thrive FHI360 Washington, DC USA; ^2^ Department of Health Promotion, Education, and Behavior University of South Carolina Columbia South Carolina USA; ^3^ School of Public Health Yale University New Haven Connecticut USA; ^4^ Food Security and Agriculture Research Triangle Institute Raleigh North Carolina USA; ^5^ USAID Integrated Nutrition Helen Keller International Kathmandu Nepal; ^6^ Alive and Thrive FHI360 Abuja Nigeria; ^7^ Alive and Thrive FHI360 Hanoi Vietnam

**Keywords:** child nutrition programmes, conceptual framework, costs | interpersonal communication, large scale, low‐ and middle‐income countries, mass media

## Abstract

Child nutrition has serious long‐term development implications. Evidence‐based frameworks and models are urgently needed to reduce deficits in infants and young children's diets on a large scale. Our paper reviews 32 publications and five impact evaluations of programmes in Bangladesh, Ethiopia, Nepal, Nigeria and Vietnam to identify what worked and why; the quality of evidence, diversity of countries and multi‐level interventions on a large scale were selection criteria. Key lessons are: the need for advocacy to prioritize complementary feeding; engagement of multiple stakeholders to reach national scale and to address diverse factors such as food access, harmful marketing of unhealthy foods and beverages, knowledge gaps, social norms and maternal self‐efficacy. Applying a behavioural science lens, monitoring intervention coverage, targeting to reduce inequalities, engaging community leaders, motivating frontline workers and leveraging mass media to reach multiple audiences worked synergistically to produce impacts at scale. Despite different contexts and dietary diversity levels at baseline, rigorous evaluations documented substantial improvements attributable to the interventions in all five countries. The expenditures incurred varied by programme complexity and showed that they are manageable if the interventions focus on priority issues, are streamlined to fit existing platforms and reach large populations. With evidence of impact in diverse contexts, an evidence‐based conceptual framework and tools for implementation, insights into how to adapt to country contexts and knowledge of what to budget, decision‐makers can invest confidently in improving complementary feeding programmes.

## Summary

- A mix of facility, community and mass media was effective in improving complementary feeding practices. A dose–response relationship was found between the channels reaching mothers and impact.
- Programme effectiveness was associated with advocacy on complementary feeding; engagement of multiple stakeholders; reduction in knowledge gaps, improved social norms and maternal self‐efficacy; use of behavioural science, monitoring coverage and complementary feeding; reducing inequalities; engaging fathers and community leaders, motivating frontline workers and leveraging mass media.
- The incremental financial costs were below one US dollar per year for reaching a mother/child and US$ 6.3 and US$ 31.4 for improving complementary feeding practices in Bangladesh and Vietnam, respectively.

## 1 Introduction

Achieving the Sustainable Development Goals and reducing child stunting, wasting and obesity depends upon improving complementary feeding (CF) (WHO 2023). CF between 6 and 24 months coincides with the peak period for risk of growth faltering and nutrient deficiencies that contribute to impaired growth, excess morbidity and mortality, and delayed motor, cognitive and socioemotional development with long‐term consequences (Black et al. 2013). Children's diets are associated with malnutrition such as underweight, wasting, micronutrient deficiencies and overweight (Arimond and Ruel 2004; Black et al. 2013; Krasevec et al. 2017; Laving et al. 2018; Schneider et al. 2020). Inappropriate CF increases risk of overweight, type 2 diabetes and disability in adulthood (Black et al. 2017). Along with continued breastfeeding, foods rich in essential nutrients need to be incorporated into children's diets and protected from pathogens and toxins, excess sugar and salt, and harmful fats, as well as prepared appropriately for the rapidly changing physiological status of infants and young children (Lutter et al. 2021). How responsively children are fed can influence their verbal, motor and psychological development (Pérez‐Escamilla et al. 2021). Taste preferences emerge early and lack of attention to CF can promote consumption of obesogenic foods and adult degenerative disease progression (Dunford et al. 2012). Achieving high prevalence of recommended CF practices among infants and young children is a challenge for many countries (UNICEF 2022). Near‐universal suboptimal CF practices in rapidly developing economies include both foods that are low in energy and nutrient‐rich ingredients as well as foods excessively high in energy, sugar, salt and trans‐fats (Pérez‐Escamilla et al. 2018; UNICEF 2021). Nutrient densities of family diets that are often fed to children particularly in low‐ and middle‐income countries (LMICs) are low in relation to the energy and nutrient requirements of children in the 6–24 months age group and often contain harmful substances such as pesticides, nitrates and nitrites, bisphenols, perchlorates, nitrates and nitrites, artificial food colours, monosodium glutamate and aspartame (Savin et al. 2022; Victora et al. 2010). CF is one of four pillars for addressing wasting, which carries a high risk of child mortality and morbidity (WHO, UNICEF, FAO, WFP, & UNHCR 2020). The need to address harmful marketing and promotion of unhealthy foods for infants and young children is urgent (Pérez‐Escamilla, Dykes, and Kendall 2023; Pérez‐Escamilla, Tomori, et al. 2023; Rollins et al. 2023; Smith et al. 2019). Countries restrict such actions through education and incentives (Boyland et al. 2022).

Despite clearly defined needs, evidence of addressing these needs through population‐level large‐scale programmes is limited (Gillespie et al. 2015), but recently, several countries have documented large‐scale CF programmes with the support of the Bill & Melinda Gates Foundation (Alive & Thrive initiative) and *Suaahara* project, funded by USAID. Our aim for this paper was to identify lessons learned to guide the next generation of programmes. Specifically, we aimed to fill gaps in current literature on the key attributes needed in CF programmes to achieve large‐scale results. We also extracted the steps and processes used by effective CF programmes to fill gaps on ‘how’ to design and implement large‐scale CF programmes. This knowledge is needed to address the continued high levels of suboptimal diets and wasting in infants and young children (UNICEF 2021, 2022; WHO 2023).

## 2 Methods

We conducted a literature review to identify CF programme‐related studies relevant for designing and implementing large‐scale programmes. The studies were selected using a search strategy for Pubmed and Corcoran reviews using the following parameters. Exclusion criteria: Small‐scale, lack of details on CF intervention content, descriptive without analysis of results and associated factors, not located in sub‐Saharan Africa/Asia. Inclusion criteria were: sub‐Saharan Africa/Asia, < 20 years, English language, full papers in the public domain, large‐scale programmes, CF interventions described in detail, rigorous programme evaluation design. To select programme models, we searched the published and grey literature, reviewed the attributes of the potential models, identified the selected programme models with positive impacts on CF and synthesized the findings related to design and implementation and finally, identified patterns and results that could inform the development of future programmes and/or contribute to the development of the conceptual framework in the paper. We identified and summarized five programme experiences of CF intervention packages in‐depth in diverse LMIC settings that had been extensively documented and rigorously evaluated for impacts on CF, inequalities, and determinants of CF practices and their costs or budget expenditures were available. We also extracted information from 32 implementation research studies on programme components to gather insights for strengthening CF programmes in other settings. Results of evaluation studies and analyses of determinants were compiled and programme details obtained from additional publications and the grey literature in organizational websites. Lessons that were related to achieving scale, coverage and impacts on CF were selected for inclusion.

The authors identified key programme design and implementation characteristics needed by decision‐makers and managers during programme development to form a matrix for synthesizing the information. The information was extracted and organized through a shared matrix with the assistance of authors who implemented various elements of the programmes in each country including design, programme content and components, programme coverage, impacts, costs and expenditures. Criteria used for selecting studies were quality of the analysis, effect size, plausibility, diversity of community contexts and programme platforms and range of interventions. Finally, we propose a framework developed from theory‐based and field‐tested evidence for CF programme planning. Two senior authors discussed the extracted information and draughted the planning framework that was shared and commented on by other coauthors before finalizing the framework. The evidence highlighted the important influence of contextual factors at the community and programme/institutional levels on CF programme results and led us to develop a checklist for adaptation of the programmes to CF programmes in different contexts. The programmes reviewed here and details on methods are summarized below.

### 2.1 Country Programmes

The country programmes shared certain characteristics, but implementation differed in response to the diverse community and programme settings. The programmes were BRAC's infant and young child feeding (IYCF) programme in Bangladesh, Government of Ethiopia's joint health and agricultural extension programme in Amhara region, Nepal's multi‐sectoral national *Suaahara* programme, Nigeria's CF programme in Kaduna and Vietnam's franchise model embedded in government health facilities. In addition to counselling of mothers and community mobilization, all programmes used national and/or regional mass media to raise CF awareness and engage multiple influential audiences including mothers. See Tables 1 and 2 for further details on programmes.

**Table 1:** Models and findings on complementary feeding programmes in Bangladesh, Ethiopia, Nepal, Nigeria and Vietnam.

Topic	Bangladesh, National 2009–2014	Ethiopia, Amhara 2015‐2018	Nepal, National 2011–2023	Nigeria, Kaduna 2019–2021	Vietnam, national 2009–2014
**Programme setting**					
Sectors and programme platforms	NGO (BRAC) used existing community service delivery and community platforms, policy and technical guidelines of Ministry of Health's Inst. of Public Health Nutrition, tech. support A&T. *Source*: Programme records	Ministry of Health policy, technical guidance, counselling, community dialogue; Ag. Extension dept. supported vegetable and poultry production. Tech. support Save the Children, A&T *Sources:* Programme records	Ministry of Health and Population FCHVs; agriculture/livestock extension Project supervisors, facilitators, Model Farmers. SCF, HKI, Jhpiego, CARE, FHI360, NTAG. *Sources:* JHU CCP https://ccp.jhu//pr objects, (Suresh et al. 2019)	Ministry of Health policy, tech. guidance, primary health care outreach for service delivery. CBOs and FBOs for community mobilization. NGO (ICWYI), tech. support A&T. *Sources:* Programme records	Ministry of Health facilities with embedded IYCF counselling centres (franchises) plus mass media; technical oversight by National Inst. of Nutrition; support by Save the Children, tech. support A&T. *Sources:* Programme records
Locations/scale	50 rural sub‐districts in six administrative regions, later expanded to 145 subdistricts; est. 1.7 million mothers of < 2 children covered by IPC. *Sources:* Programme records	10 districts (*woredas*) in 3 western zones of Amhara region, rural communities, families mostly from Ethiopian Orthodox Church. *Source*: Programme records	41 underserved districts in Nepal reaching out to over 900,000 HH with pregnant and lactating women and < 2 years children *Source*: Facts sheets	10 towns in Kaduna state, northern Nigeria, with a 60% urban population, a mix of religions and ethnic groups. *Source*: Programme records	781 social franchises in govt. centres, 15/63 provinces at multiple levels (provincial, district, commune centres) expanded to > 1000 franchises in 18 provinces. *Sources:* Programme records
**Community context**					
Mothers' profile	‐ Age = 27 years ‐ Schooling = 6.5 years ‐ Occupation = housewife 94%. *Source*: Menon et al. (2016)	‐ Age = 28 years ‐ Schooling = 2.3 years ‐ Occupation = housewife 25%. *Source*: Kim et al. (2019)	‐ Age = 27 years ‐ Ethnic/socially excl. groups = 43% ‐ 2 lowest wealth quintiles = 52%. *Source*: Frongillo et al. (2024)	‐ Age = 25 years ‐ Schooling = primary, secondary 33% each ‐ Occupation HW 47%. *Source*: Flax et al. (2022)	‐ Age = 29 years ‐ Schooling = 9 years ‐ Occupation = 56% farmers. *Source*: Rawat et al. (2017)
Child dietary diversity	MDD = 31%. *Source*: Menon et al. (2016)	MDD = 5%. *Source*: Kim et al. (2019)	MDD = 43%. *Source*: Frongillo et al. (2024)	MDD = 61%. *Source*: Flax et al. (2022)	MDD = 74%. *Source*: Rawat et al. (2017)
Barriers to desired CF practices	‐ Poor maternal and family CF knowledge of all aspects ‐ Low self‐efficacy in feeding recommended foods, amounts ‐ Perception of poor appetite in 6–23 months children, so effort not made to feed ‐ 25% of family had low access to food. *Sources:* (Menon et al. (2016); Rasheed et al. (2011))	‐ Knowledge of CF lacking in mothers, family members, HEWs and HDATLs and agriculture extension workers ‐ Religious fasting periods (typically for 100–110 days/year) followed extensively when no animal foods are prepared; also restrict cooking, types of foods. *Sources:* Kim et al. (2019)	‐ Knowledge of CF in mothers, family members, health workers, community volunteers ‐ CF norms, gender norms linked to mothers' workload ‐ Access to nutrient‐ rich foods, HH food security. ‐ Quality of CF counselling. *Sources:* Dickin et al. (2021), Thapa et al. (2024)	‐ Low knowledge of age of introduction, nutrients in food, amounts needed in parents ‐ Fruits/vegetables are considered ‘food for others’; grown to sell, not kept ‐ ASF given to adults/parents, low rural availability ‐ Mothers' low access to specified foods, cost is a barrier. *Source*: Allotey et al. (2022), Flax et al. (2022)	‐ Good knowledge but no FLW counselling protocol or accountability for CF ‐ Low facility attendance after immunizations. ‐ Premature introduction, low nutrient density ‐ Contradictory commercial ads. *Sources:* Nguyen et al. (2016, 2014), Nguyen, Menon, et al. (2011)
Influential persons and information *sources*	Local doctors, older women in family, peers, fathers (for food procurement). *Sources:* (Menon et al. 2016; Rasheed et al. 2011)	HEW, mothers in law, husbands (food procurement), peers, doctors/nurses. *Sources:* Kim, Mwangi, and R (2015)	Family members, FCHVs, govt. workers across sectors. *Source*: 2017–2020. usaid. gov/Nepal/fact sheets/Suaahara	Family members especially fathers (purchase of food); CBOs, FBOs, FLWs. *Sources:* Flax et al. (2022), Reboot and Picture Impact (2017)	Husbands, health workers, doctors, peers, commercial marketing. *Sources:* Nguyen, Manohar, et al. (2011), Nguyen, Menon, et al. (2011)
**Intervention design** a					
Counselling and education content	Timely intro., continue BF, dietary diversity (ASF, DGLV, pulses), number of meals, ill child diet, handwashing; responsive feeding with ECD content (verbal, foods/colours; motor, self‐feeding). *Source*: Programme reports, tools	Dietary diversity using DGLV and eggs from home produce added to usual meals; continued BF, feeding during/after illness, meal frequency and amount, handwashing. *Source*: Programme reports, tools	Timely introduction of CF, dietary diversity, meal frequency and feeding during child's sickness; handwashing. *Sources:* Cunningham et al. (2017), Frongillo et al. (2024)	Adding two nutrient dense food varieties (ASF, beans/pulses, fruits/vegetables) to usual food, continued BF, feeding sick child/poor appetite. Fathers: funds/food, remind to feed key foods daily, accompany child at meals. *Source*: Tools, programme reports	Timely introduction of water/liquids/solids, age for starting specific foods, feeding methods, recipes, benefits or adequate CF diversity including animal *source* foods, quantity, feeding the sick child, GMP, schedule of visits Source: Programme reports, tools
Providers of IPC	BRAC frontline workers (FLWs): salaried nutrition‐focused *Pushti Kormi* (PK), health‐focused *Shasthya Kormi* (SK) and performance‐based incentivized volunteers *Shasthya Sebika*. *Source*: Programme reports	Salaried government health extension workers (HEWs), health development team leaders (HDTLs, community health volunteers) and agricultural extension workers *Source*: Programme reports	Salaried project field staff trained to conduct home visits and facilitate community events such as food and handwashing demonstrations, with incentivized FCHVs. *Source*: Cunningham et al. (2021)	Salaried outreach workers (CHEWs) conducted home visits and used immunization visits for IPC with parents. Digital media platforms sent weekly messages to fathers by text and voice format. *Source*: Programme reports	Salaried health care providers at primary health centres held individual and group sessions on CF, accompanied by growth monitoring and promotion. *Source*: Programme reports
Community mobilization	Volunteers promoted CF practices for fathers; field managers facilitated community events focused on CF behaviours; FLWs demonstrated amounts of nutrient‐dense family foods, fathers' role in supporting CF *Source*: Programme reports	‐ Priests and leaders from the Ethiopian Orthodox Church delivered sermons about dietary diversity, child feeding during fasting periods ‐ ‘Community conversations' on CF conducted by CBOs ‐ Dramas enacting ideal CF *Source*: Programme reports	‐ Health, nutrition, family planning, water, sanitation and hygiene, backyard poultry and food production messages through govt. and project workers through women's health group meetings. *Source*: USAID factsheet, Frongillo et al. (2024)	Fathers reached through religious leaders, CBO community gatherings/town hall meetings, mobile phone contacts, visits to fathers from CHEWs, materials for fathers and religious/traditional leaders to guide meetings. *Source*: Programme reports	CM was operated by village health workers who visited households of women with children aged < 24 month to ‘invite’ mothers to attend counselling services and provide women with basic IYCF messages. *Source*: Programme reports
Media and public education	3 TV and 3 radio spots on age‐specific amounts, dietary diversity, poor appetite broadcast nationally; in media‐dark (no electricity) areas, generators hauled and TV/radio shows plus dialogue conducted with communities *Source*: Programme reports	12‐episode regional broadcasts per season through 3 radio stations on best IYCF practices; radio dramas, spots and magazine formats. Media‐dark areas received the same through mobile vans and drama shows *Source*: Programme reports	The mass media edutainment radio programme, titled *Bhanchhin Aama* (Mother Knows Best), covers a wide range of IYCF and health practices, use of multiple channels. *Source*: Cunningham et al. (2021), Suresh et al. (2019)	1 TV and 1 radio spot was broadcast in the local language with visuals adapted for Kaduna on dietary diversity and cognitive benefits, role of family highlighted; talk shows covered Q&A and lectures on IYCF in the local language. *Source*: Programme reports	1 spot focused on complementary feeding, and 1 spot promoted the use of franchise services, loudspeaker announcements, billboards and bus wraps, website for Q&A, fathers' competitions. *Source*: Programme reports
**Coverage and impact** b					
Coverage reached through IPC, community dialogue	92% received IPC in the last 6 month from nutrition worker and 89% from a community volunteer; exposure to CM session, 41% *Source*: Menon et all. (2016)	32% of mothers recalled IPC on CF at a health post, 36% recalled agricultural extension messages, 34% saw a food demonstration and 21% sermons that included CF. *Source*: Kim et al. (2019)	35% of mothers recalled at least 1 exposure to IPC/CM/MM 1 year after starting. Scale‐ up in > 50% districts in Nepal, while targeting of disadvantaged groups. *Source*: Suresh et al. (2019)	11%–26% fathers recalled exposure to at least 1 channel and 12%–21% mothers. 16% IPC (fathers), 21% IPC (mothers). COVID‐19 restrictions closed IPC for 3/12 months programme. *Source*: Flax et al. (2022)	42% mothers were ever exposed to IPC at a franchise facility (39.3% in the last 6 month); 1.3 mean number of visits in the previous 6 month (approx. 50% of target). *Source*: Rawat et al. (2017)
Mass media/social media coverage	73.4% of mothers in intervention areas and 67.2% in non‐ intervention areas recalled a CF TV spot. *Source*: (Menon et al. (2016)	35.4% of mothers recalled listening to the Sebat Mela radio programme. *Source*: Kim, Nguyen, et al. (2018)	51.3% of mothers reported listening to the *Bhanchhin Aama* (Mother Knows Best), radio programme. *Source*: Cunningham et al. (2021)	23%–26% of fathers, 16%–21% mothers recalled mass media messages from TV or radio. *Source*: Flax et al. (2022)	36.4% of mothers recalled the message on CF from TV or social media and other mass media channels. *Source*: Rawat et al. (2017)
Impact on CF practices	MDD, MMF and MAD improved by 16.3, 14.7 and 22.0 pp respectively, in the programme areas reaching 50.4% for MAD, 63.8% for MDD, 75.1% for MMF at endline. *Source*: Menon et al. (2016)	MDD and MAD increased at endline to 24.9% and 18.2%, respectively. Stunting declined (DDE: −5.6 pp; *p* < 0.05) decreasing from 36.3% to 22.8% in the programme areas. *Source*: Kim et al. (2019)	MDD improved from 43% at baseline to 68% at endline, introduction from 12% to 32%, sick child feeding from 13% to 35%, MMF from 67% to 84% and MAD from 32% to 55%. *Source*: Frongillo et al. (2024)	MMF increased from 58% to 73%, intake of fish by children from 36% to 44% and eggs from 8% to 20%, MDD remained around 65%. *Source*: Flax et al. (2022)	MDD (DDE: 6.4 pp) and MAD (DDF: 8.0 pps) improved among mothers who attended IPC counselling at least once compared to those from the control or non‐intensive group. *Source*: Rawat et al. (2017)
Impact evaluation designs	Cluster‐randomized, cross‐sectional surveys (*n* = ∼600 and 1090 children 6–23.9 months at baseline [2010] and *n* = ∼500 at endline [2014]); difference‐in‐ difference impact estimates (DDEs)	Cluster‐randomized, cross‐sectional surveys, children aged 6–23.9 months[*n* = 2646 at baseline (2015) and *n* = 2720 endline (2017)]; DDEs, dose‐ response and path analyses for plausibility	Changes compared in intervention and comparison districts over 10 years; *n* = 2040 HH at baseline in 2012 and *n* = 2480 HH at endline; intent‐to‐ treat regression analysis	Cross‐sectional population‐based surveys of fathers and mothers with a child aged 6–23 months, regression models compared population estimates at baseline (2019) *n* = 497 and endline (2020) *n* = 495.	Cluster‐randomized, cross‐sectional surveys *n* = ∼500 children 6–23.9 months at baseline (2010) and endline (2014); DDEs for intent‐to‐treat and modified per‐protocol analyses of mothers who received IPC at least once

Abbreviations: A&T, Alive & Thrive initiative; ASF, animal source food (fish, eggs, meat); BF, breastfeeding; BRAC, Bangladesh national NGO; CBO, community based organizations; CF, complementary feeding; CM, community mobilization; CHEW, community health extension worker; CM, community mobilization; DDE, difference in difference; DGLV, dark green leafy vegetables; ECD, early childhood development; FBO, faith‐based organization; FCHV, Female Community Health Volunteers; FLW, frontline worker; HH, households; HEW, health extension worker; HDATL, health development army team leader; ICWYI, I Care Women and Youth Initiative (NGO); IPC, interpersonal communication; IYCF, infant and young child feeding; MDD, minimum dietary diversity; MM, mass media; mo, months; NGO, non‐govt. organization; NTAG, Nepali Technical Assistance Group; PHC, primary health care; pp, percentage points; RCT, randomized controlled trial; SBCC, social and behaviour change communications.

^a^ For detailed information on field operations and training of frontline workers see, https://www.aliveandthrive.org/sites/default/files/attachments/Bangladesh-Community-Implementation-Manual-Jan_2014.pdf, https://image.savethechildren.org/alive-and-thrive-franchise-toolkit-ch11044545.pdf/1c58643rp1txxc02o572uckwje266qja.pdf, https://thecompassforsbc.org/sbcc-tools/suaahara-training-guidelines-and-participant-handbooks.

^b^ Stunting declined in Ethiopia in programme areas relative to nonprogram areas. In Bangladesh, Nepal and Vietnam stunting declined almost equally during the programme period in programme and nonprogram areas.

**Table 2:** Summary of studies on complementary feeding programme implementation research.

Author; location	Study design	Findings	Programme lessons
**Priority CF practices and determinants**			
Nguyen, Manohar, et al. (2011), Nguyen, Menon, et al. (2011); Vietnam	Review existing data, organize evidence into conceptual framework for systems strengthening, policies and IPC	Enabling government policies, support from NGOs, and family members could be key in improving CF practices. Needed to engage multiple stakeholders in family, health system and the private sector. To identify priority CF behaviours for programme focus, existing data and information can be valuable, but new formative research may also be needed and baseline survey data can provide prevalence of poor diets and barriers to shape programme design (Nguyen, Manohar, et al. 2011; Nguyen, Menon, et al. 2011).	Premature introduction of foods, liquids and feeding low nutrient quality foods and liquids. Barriers: knowledge gaps (FLW, mothers) incorrect beliefs among mothers.
Rasheed et al. (2011); Bangladesh	Semi‐structured *n* = 42 mothers, observations *n* = 21 child–caregiver pairs, TIPs *n* = 94; KII *N* = 6, FGDS = 10, 1‐day diet recall *n* = 195	Need to focus on adequate amounts of solid foods and food diversity, particularly ASF. HH food availability necessary but not sufficient. Perceived child‐appropriateness/benefits are key drivers, mothers' knowledge, skills, perceptions, social norms. Suggest choices based on home meals, engage family to procure food (men), mothers' time (family members)—keys to success (Rasheed et al. 2011).	Mothers unaware about how to address critical barriers (lack of time, child refusal, food choices); FLW skills, family dialogue are gaps
Cresswell et al. (2017); Burkina Faso	HH survey (*n* = 2288 mothers with live birth < 1 year) multi‐variable analysis	Extreme lack of energy and nutrient containing foods given to children below 1 year. Low knowledge and practice of CF. < 2% of infants 6–11 months had a minimum acceptable diet; a third of infants 6–11 months received liquids and 10% breastmilk and water only (Cresswell et al. 2017).	Poor knowledge, social norms and seasonal effects drive huge dietary gaps in the 6–11 months age group
Nguyen, Frongillo, et al. (2019), Nguyen, Kim, et al. (2019); Bangladesh	HH surveys in 2014 and 2016 (post‐programme) (*n* = ∼2000). Multi‐var. analysis tested differences in networks, diffusion, norms; pathways (exposure‐practices)	Mothers' social networks, diffusion of information and social norms were associated with improved IYCF practices; MDD was associated with larger social networks and continued diffusion (OR: 1.5–2.2). NGO continued IPC 2 years after programme ended as part of its routine Essential Health Care programme in expanded areas, though less intensively. Message diffusion through other health workers and family members, and govt. broadcasting of mass media after external support ended drove continued coverage (Nguyen, Frongillo, et al. 2019; Nguyen, Kim, et al. 2019).	Social networks are key to achieve and sustain IYCF practices; CHWs play important role; even less intensively implemented actions can support diffusion of IYCF practices.
**Influential persons**			
Rasmi et al. (2013); Bangladesh	FLW knowledge (*n* = 100), observed CF (*n* = 37), trial/adoption (*n* = 64), training reviewed	Key facilitators of adopting practices included family support and availability of resources. Lack of time, maternal and family perceptions of age‐appropriate feeding, lack of resources perceived as barriers. Management issues: role of paid and volunteer staff and their effective utilization (Rasmi et al. 2013).	Family support was identified as critical for adoption of CF practices through use of a PIP analysis
Reboot and Picture Impact (2017); Nigeria	Identified persons, ecosystems, channels of communication and messages through qualitative methods to improve IYCF	Close female relatives, e.g., mothers‐in‐law in Yoruba culture, mothers in Hausa culture and neighbours in urban settings. Health care systems are the second key sphere of influence (formal and informal). Fathers have the potential to be either a positive or negative influence. Daycare providers, community and religious leaders are sources of information, have regular interactions, willing to be leveraged (Reboot and Picture Impact 2017).	Grandmothers plus male persons with influence should be engaged to address food access, convenience, support for CF practices
Warren et al. (2020); Bangladesh	20 subdistricts randomly assigned to programme (*n* = 4281 HH) or nonprogram (*n* = 4284 HH) groups; multivar. analysis tested differences in expenditures, MDD	Expenditures on ag. produce e.g., eggs and flesh foods increased more in intensive areas than in non‐intensive areas by 53 and 471 *taka*/month, respectively. HH food expenditures increased more in intensive areas by 832 *taka* (*p* = 0.02), nonfood expenditures did not differ. Women's employment and control of income increased more in intensive areas; jewellery ownership decreased more by 23 percentage points. Higher expenditures on food groups were reflected in higher consumption by women and children (Warren et al. 2020).	Counselling fathers with tailored interventions improved HH expenditures on recommended nutrient‐rich foods (eggs, flesh foods) and MDD
Allotey et al. (2022); Nigeria	HH surveys (*n* = 495) parents; logistic regression to examine associations	When fathers provided multiple CF support actions (i.e., more than the traditional procurement of food), MMF, MAD and feeding children fish improved. A majority of parents agreed with a broader support role for fathers (Allotey et al. 2022).	Fathers' role beyond food procurement is acceptable and can improve CF practices
**Key sectors**			
Sanghvi et al. (2013); Bangladesh, Ethiopia, Vietnam	Situational assessments, stakeholder meetings, formative studies (HH, FLW, mothers); monitoring	Health system needs for CF include explicit protocols, fit‐for‐task human resource allocations, basic and refresher training, job aids, supportive supervision and FLW incentives/motivation, monitoring data. Leadership, financing, partnerships, logistics support are essential to support scaling up in diverse communities. Ongoing learning, feedback can inform programme revisions for coverage/quality (Sanghvi et al. 2013).	NGO and govt. health services can deliver counselling services in communities and facilities with stronger systems
Nguyen et al. (2014); Vietnam	Franchise management interviews (*n* = 12), FLW (*n* = 120), obs. (*n* = 160), HH surveys (*n* = 2045)	Health franchise utilization increased from 10% in 2012% to 45% in 2013 but did not reach target of 9–15 contacts; 12% of 6–24 months completed target visits. Barriers: lack of PHC attendance, low coverage of demand generation interventions, mothers' work conflicts, busy schedule, unaware of franchise; but quality of franchise services improved (Nguyen et al. 2014).	Challenge: delivering IPC to caregivers of 6–23‐month olds due to low attendance, limits use of health centre‐based services
Hirvonen and Wolle (2019); Ethiopia	Secondary analysis of existing surveys from ag., social protection, health and dietary recall studies	NSA priorities: Legumes, nuts in Afar (not widely consumed among children, available in rural markets, supply stable and affordable) (Hirvonen and Wolle 2019). Eggs and vit. A rich fruits and vegetables in Amhara; legumes and nuts, eggs or vit. A rich fruits and vegetables in Oromia; eggs, and legumes and nuts in SNNP; lack of rainfall and absence of irrigation in Somali limit choices but eggs have potential; eggs in Tigray	Existing ag. sector data on affordability, access, consumer choices, production, market availability can identify NSA for key CF foods
Frongillo (2^020^); Bangladesh, Ethiopia, Vietnam, Mexico	Assessments and evaluations of A&T CF programmes, and Mexico's scripted radio messages, and cash transfer programme	Multiple sectors needed; partnerships and alliances are key; well‐grounded understanding from research should inform a public health strategy for CF; use scalable programme platforms and elements in existing systems where possible; conduct monitoring, learning and evaluation throughout; because of the multi‐faceted drivers of CF behaviours involved, strategically select and focus on critical sectors (Frongillo 2020).	Jointly plan with multiple sectors and partners for scale and sustainability from the start
**Platforms for delivering interventions**			
Nguyen et al. (2014); Vietnam	Process assessment 12 months post‐launch: facilities observation (*n* = 32), staff (*n* = 96), obs. (*n* = 137), exit int. (*n* = 137)	Utilization of health franchises was low (10% of target). A higher proportion of pregnant women utilized franchise services (48.9%), compared with mothers with children 6–23.9 months (1.4%). Franchise users praised problem solving related to CF. Service delivery was high, but population impact depends upon utilization of facilities. CF improved with ≥ 1 visit (Nguyen et al. 2014).	CHWs were mobilized to generate more visits for CF counselling; high quality and problem‐ solving increased use
Nguyen et al. (2016); Vietnam	Mothers of < 2 years (*n* = 1008), health staff (*n* = 60); regression analysis to identify drivers of utilization	Among health system factors, good counselling skills (PR: 1.3–1.8) was key; additional 25% of the population would have achieved the minimum number of visits if exposed to three different demand‐generation strategies, and could further increase to 49% if the health staff had good counselling skills and low work pressure (Nguyen et al. 2016).	Demand generation to use CF counselling and good quality counselling important for facility‐based counselling
Sanghvi et al. (2017); Bangladesh, Malawi, Peru, Zambia	Evaluations of 4 scaled up programmes with improved dietary diversity examined for commonalities and differences in their respective designs and implementation	Bangladesh and Peru used social behaviour change through NGO and govt. health services and food access was not an issue; Malawi and Zambia reduced barriers to food access and engaged the agricultural sector plus health services for promotion of recommended CF practices. The programmes leveraged relevant platforms, e.g., CHW, community events, mass media, women's empowerment, handwashing materials, ag. inputs, national/subnational advocacy, alliance building and multisectoral sectoral coordination (Sanghvi et al. 2017).	Engaging sectors based on assessed need for addressing specific barriers identified through research and effective roll‐out resulted in improved dietary diversity
Kim, Roopnaraine, et al. (2018); Bangladesh	HH surveys (*n* = 2000), interviews (*n* = 251) with mothers/HH members; logistic regression, and transcripts analysed	Exposure ranged from 36% to 62% across 6 TV spots, with comprehension ranging from 33% to 96% among those who viewed the spots. Synergies found between IPC and mass media. Three direct narrative spots showed correct message recall and strong believability, identification and feasibility of practicing the recommended behaviours. Metaphorical and indirect narrative style were not well understood (Kim, Roopnaraine, et al. 2018).	Well‐designed mass media that reaches key influential persons in families/communities can be key to improving IYCF at scale
Nguyen et al. (2021); Vietnam	Cross‐sectional survey in 3 provinces, communes with intervention (*n* = 551), without inter‐ vention (*n* = 559)	IYCF support groups in remote villages across nine provinces reached > 30,000 pregnant women and mothers with children aged 0–23 months. Regression models showed that living in intervention communes was associated with higher odds of minimum acceptable diet (OR: 1.51; 95% CI: 0.98, 2.33) compared to those living in comparison communes (Nguyen et al. 2021).	Community support groups with trained facilitators in underserved areas can improve CF practices
**Scale strategies and budgetary needs**			
Sanghvi et al. (2013); Bangladesh, Ethiopia, Vietnam	Situational assessments, stakeholder consultations, formative research, HH and FLW surveys, programme monitoring	To scale up IPC services, countries used different approaches with differing results: a standardized franchise model for IYCF counselling services in Vietnam in government health facilities; counselling delivered at the doorstep by incentivized NGO volunteers in Bangladesh; and in Ethiopia, facility‐based and outreach by government health extension workers with support from volunteers. Strengths and weaknesses in existing platforms transferred to CF results (Sanghvi et al. 2013).	More extensive pilot testing and flexibility to change approaches may have improved results; 3 key factors: service quality, plan execution, utilization by mothers
Gillespie et al. (2015); Bangladesh, Nepal, Mexico	Literature review including 36 frameworks and 4 case studies to compare programme content, scale strategies, results, challenges; identify success factors	Programmes: an Ag. plus SBC Bangladesh project, A&T's Bangladesh IPC through NGO (BRAC), Mexico govt.'s social protection, Nepal's IFA supplementation. Key elements for effective scaling up identified as: (1) clear vision/goal; (2) streamlined intervention; (3) platform to leverage for scale; (4) motivators/catalysts for scale, e.g., champions, and incentives; (5) locally tailored, (6) capacity; (7) financing; (8) governance and (9) monitoring, learning and accountability (Gillespie et al. 2015).	Political commitment to the nine key elements identified will be crucial to achieve sustained results at scale. A scaling up framework is presented.
Sanghvi et al. (2016); Bangladesh	Situation analysis, formative research, e.g., market survey, semi‐structured interviews, food attributes exercise observations, media scan (Nielsen); pilot testing; process evaluation; final evaluation.	High reach platforms engaged; small, achievable actions for key audience segments identified through rigorous testing; underlying behavioural determinants addressed; 60%–90% of the priority groups reached through repeated IPC and media contacts. Community volunteers received monetary incentives for each mother in their areas who practised recommended behaviours; 8.5 million mothers benefited over 4 years. Scale‐up was facilitated by streamlining tools and strategies, mass media, govt. branding, use of existing community‐based platforms, advocacy and readiness of stakeholders who witnessed stagnant national IYCF indicators despite baby‐ friendly hospital initiative and training (Sanghvi et al. 2016).	Existing supportive govt. policies, mass media and nationwide mainstreaming of IPC through multiple stakeholders achieved scale. Challenges: frequent changes in key counterparts, dilution due to new priorities.
Cunningham et al. (2017); Nepal	HH surveys (*N* = 480) to assess relevant health, nutrition, and water, sanitation and hygiene (WASH); also assessed inequalities; multivar. regression models for impacts	After only 2 years of full programme intervention, large differences were found in exposure, knowledge and some practices between comparison and intervention groups for maternal and child health and nutrition, as well as WASH. Progress was achieved in difficult to move child nutrition indicators, such as appropriate sick child feeding. Utilization of existing community‐based cadres of workers and supplemental systems capacity strengthening across multiple sectors can achieve scale in integrated programme interventions. If inputs are targeted to disadvantaged communities, nutrition programmes can also reduce inequalities (Cunningham et al. 2017).	Large‐scale multi‐sectoral, integrated interventions can improve nutrition‐related knowledge and practices and reduce inequities if targeting of interventions is well planned and executed.
Choo et al. (2024); Nepal	Total and unit costs calculated from expenditure data combined with economic costs assessed using in‐depth interviews and focus group discussions in four representative districts	The average annual total cost was US$908,948 per district, with economic costs accounting for 47% of the costs; out‐of‐pocket costs would be lower. The annual unit economic cost was US$132 per participant reached. Annual economic costs ranged from US$152 (mountains) to US$118 (plains) per programme participant. Personnel (63%) had the largest input cost, community events (29%), household counselling visits (17%) were the largest activity costs. Implemented in 42 of Nepal's 77 districts (389 municipalities), the programme successfully targeted disadvantaged families and reached > 2 m. mothers and children, and nine million household members.	Multisectoral nutrition programmes can be costly, due to inclusion of volunteer and participant opportunity costs. This study provides costs of a scaled‐up multisectoral nutrition programme
Sanghvi et al. (2024); Ethiopia, Bangladesh, Vietnam	Incremental financial costs of strengthening three large‐scale CF programmes calculated retrospectively from expenditure records in Ethiopia, Bangladesh and Vietnam; reach and impact evaluated through RCTs	The programmes reached between 1 and 2.5 million mothers and children annually per country and 0.7 to 1.6 million persons who were influential in supporting mothers and achieving scale. The incremental financial costs were between $0.9 to $1.6 per mother and child reached. The largest cost component was counselling of mothers; mass media costs were lower. For the additional number of children who achieved an acceptable diet, the annual incremental financial cost was $6.3 to $34.7 per child benefited. *Sources:* Incremental Financial Costs of Strengthening Large‐Scale Child Nutrition Programmes in Bangladesh, Ethiopia and Vietnam (unpublished); reach and impact (Kim et al. 2016; Menon et al. 2016; Rawat et al. 2017).	CF programmes can be affordable for LMICs if gaps in existing programmes are selectively strengthened and large scale is reached. Costs of treating a malnourished child are several‐fold higher
**Factors influencing intervention performance and sustainability**			
Kim, Ali, et al. (2015), Kim, Mwangi, and R (2015); Ethiopia	Qualitative: supervisors, HEWs, volunteers (*n* = 54), mothers of < 2 (*n* = 60); surveys: FLWs (*n* = 504) and mothers (*n* = 750)	Training coverage and quality were high. Job aids used regularly by most supervisors and HEWs, but only 54% of volunteers in Tigray region and 39% in SNNPR region received them. Quality of service delivery and aided recall of key messages in their clients was lower in volunteers. Although FLW supervision exposure was high, content and frequency were irregular. Need to intensify community‐level delivery (Kim, Ali, et al. 2015; Kim, Mwangi, and R 2015).	Strong fidelity in training higher level staff is not enough if tools and messages are not cascaded down to reach volunteers/mothers
Nguyen, Frongillo, et al. (2019), Nguyen, Kim, et al. (2019); Bangladesh, Vietnam	Multiple regression analyses for coverage differences and links to IPC performance was done using path analyses based on HH and FLW surveys	Exposure to single and combined interventions led to variable FLW IPC performance and mothers' IYCF knowledge. Systems strengthening elements for IPC included protocols, specialized training, job aids and regular supportive supervision; they were combined with strategic placement of mass media. FLW performance linked to end‐user‐level outcomes such as higher service received (*β* = 0.12–1.04 in Bangladesh and 0.11–0.96 in Vietnam), and maternal knowledge (*β* = 0.12–0.17 in Bangladesh and 0.04–0.21 in Vietnam (Nguyen, Kim, et al. 2019).	FLW knowledge, motivation and improved service delivery can be achieved through effective training, supportive supervision, mass media exposure
Epstein et al. (2019); Bangladesh	FLW survey (*n* = 74), caregiver survey (*n* = 232), counselling observations (*n* = 232).	Counselling compliance was significantly and positively associated with both health worker self‐efficacy and technical knowledge but only marginally associated with MDD. CF behaviour change is multifactorial and affected by factors including FLW self‐efficacy plus household factors, that go beyond quality of care and counselling (Epstein et al. 2019).	Importance of addressing FLW self‐efficacy in improving MDD, not only knowledge
Kim et al. (2020); Bangladesh, Ethiopia, Vietnam	Endline surveys (*n* = 3720 mothers with children aged < 2 years). Multivariable regression models were used for analyses.	Contacts varied: 8 visits in the last 6 months in Bangladesh, 2 visits in the last 3 months in Ethiopia, and 1 visit in the last 6 months in Vietnam. Near‐monthly visits = 2–3 times higher odds of IYCF practices in Bangladesh and Ethiopia. Frequent, skilled FLW contacts with mothers combined with CM and public education improved practices in Bangladesh, Ethiopia and Vietnam at scale (Kim et al. 2020).	No specific number of IPC contacts is required for results, but more exposures through CM, MM may be needed if IPC is infrequent
Kim, Nguyen, et al. (2018); Bangladesh	Repeated cross‐ sectional surveys at baseline (*n* = 2188), endline (*n* = 2001) and follow‐up (*n* = 2400) 2 years after cessation, in the same communities	In intensive areas, exposure to IPC decreased slightly between endline and follow‐up but remained high (88.9%–77.2%); exposure to CM activities decreased significantly (29.3%–3.6%); and MM exposure was mostly unchanged (28.1%–69.1% across 7 TV spots). IYCF indicators declined from endline to follow‐up (2 years after programme ended) but remained higher than baseline. Large differential improvements of 12–17 percentage points in programme areas between baseline and follow‐up remained for timely introduction of foods, intake of iron‐rich foods (Kim, Nguyen, et al. 2018).	After external support stopped, impact on CF remained for timely CF, iron‐rich foods though intervention intensity declined slightly. Need to plan early for sustaining programme activities
Suresh et al. (2019); Nepal	HH survey < 5 years child (*n* = 3935); regression models to test associations with exposures	Positive associations were found between mass media and meeting MDD (OR: 1.38; *p* = 0.04) among children aged 6–23 months and between mass media and MDD scores (*b* = 0.15; *p* = 0.01) among children aged 2–5 years. Exposure to multiple platforms had the strongest association with child (aged 2–5 years) MDD scores (*b* = 0.41; *p* < 0.001) (Suresh et al. 2019).	A multi‐platform intervention package with IPC, CM, MM is effective in improving child dietary practices
Moucheraud et al. (2020); Bangladesh and Vietnam	Interviews and focus groups *n* = 218 stakeholders; surveys, *n* = 668 health workers, *n* = 269 service observations in former programme/non‐ programme areas	Stakeholders perceived declines in policy and advocacy activities, social mobilization activities and mass media campaigns ‐ counselling activities were institutionalized and continued in Bangladesh and Vietnam. Quantitative data show a persisting modest intervention effect: health workers in intervention areas had significantly higher child feeding knowledge. Competing priorities diluted the focus. Some components declined in frequency, quality, coverage, or were eliminated (Moucheraud et al. 2020).	Sustaining multi‐ component activities and integration into existing programmes need stable finances & staff, champions within delivery systems to sustain key elements
Glenn et al. (2021); Bangladesh	Qualitative study; seven focus groups (*n* = 43 respondents) with CHW supervisors	Removal of financial incentives led to CHWs' fewer and lower quality visits, CHW attrition and substitution for other income‐ generating activities. Volunteer CHWs at poorest levels cannot afford to work without compensation; systems strengthening should finance proven CHW functions through salaries and/or incentives (Glenn et al. 2021).	Essential role of CHWs in delivering IPC at scale and what is needed to maintain coverage and quality
Moucheraud et al. (2022); Bangladesh	Cross‐sectional surveys of FLWs at baseline (*n* = 290), endline (*n* = 511) and post‐endline 2 years after ending (*n* = 600)	Number of IYCF topics discussed during IPC decreased 2 years after ending. Refresher trainings were protective of service delivery. Between baseline and endline, the intervention increased health workers' knowledge and this improvement persisted to post‐endline, suggesting a sustained programme effect on knowledge. Job satisfaction and readiness both saw improvements among workers in intervention areas during the project period (baseline to endline) but regressed to a similar level by post‐endline (Moucheraud et al. 2022).	FLW knowledge gain persisted 2 years after programme support ended, but motivation to deliver services e.g., job satisfaction and readiness did not persist similarly
Sanghvi et al. (2024); Bangladesh, Ethiopia, Vietnam	Endline surveys re‐analysed for inequalities using Erreygers' index	Coverage, and CF practices were better in intervention areas, but coverage and practices favoured the better‐off and more educated mothers. In all 3 countries, only 5–6 variables out of 16 that were measured favoured disadvantaged mothers or were neutral (Sanghvi et al. 2024).	Inequalities found in CF practices despite programme; feasible messages, targeted delivery are needed

Abbreviations: A&T, Alive & Thrive initiative; ASF, animal source foods; CF, complementary feeding; CHW, community health workers; CM, community mobilization; DID, difference in difference; DDE, difference in difference estimates; FBO, faith‐based organization; FGD, focus group discussion; FLW, frontline worker; HEW, health extension worker; HKI, Helen Keller International; IPC, interpersonal communication; IYCF, infant and young child feeding; KII, key informant interview; MAD, minimum acceptable diet; MDD, minimum dietary diversity; MMF, minimum meal frequency; *n*, sample size; NSA, nutrition sensitive agriculture; PHC, primary health centre; PIP, programme impact pathway; RCT, randomized controlled trial; SNNP, Southern Nations Nationalities and Peoples region; TIPs, trials of improved practices.

### 2.2 Design Process and Programme Components

We described the programme interventions and obtained supplementary information from country implementers. The co‐authors were directly involved in design and implementation. We identified journal articles and grey literature on the programme design and operational components for the five programmes and reviewed tools and materials for details of programme content.

Coverage and impacts on CF practices: External agencies evaluated the programmes using rigorous designs, and they have published the results in journal articles (Flax et al. 2022; Frongillo et al. 2024; Kim et al. 2019; Menon et al. 2016; Rawat et al. 2017). Most countries used a 6‐month recall period for mothers to ask about coverage of interpersonal communication (IPC), community mobilization and mass media, except Nigeria that used 30 days. CF practices were measured using standard WHO/UNICEF indicators for IYCF practices in all countries (WHO 2010a).

### 2.3 Costs and Expenditures

Three of the five programmes provided data on the costs and/or expenditures. The *Suaahara* project in Nepal conducted a comprehensive economic and financial costing of the programme (Choo et al. 2024). Programmes in Bangladesh and Vietnam conducted a financial expenditure analysis of incremental out‐of‐pocket costs based on accounting records (submitted for publication).

### 2.4 Inequalities in CF Practices and Programmes

Data from endline evaluations of the programmes in Bangladesh, Ethiopia and Vietnam were used to conduct analyses with concentration indices to determine how well interventions performed regarding inequality or socioeconomic disparities in coverage and CF practices (Sanghvi et al. 2024). The Nepal programme compared CF impacts across wealth quintiles (Frongillo et al. 2024).

### 2.5 Proposed Planning Framework

Based on information from impact studies and multivariable analyses on barriers, programme platforms, interventions coverage, impacts and associated factors, we developed a conceptual framework for guiding CF programme planning and a checklist on how programme implementation strategies can adapt to local conditions.

### 2.6 Ethics Statement 

No new data were collected for this study; we used secondary data sources and published papers with citations that had declared ethical clearance from institutional boards previously.

## 3 Results

With the aim of informing future large‐scale CF programmes based on lessons learned on design and implementation, we present descriptions of the programme models and lessons learned from evaluation results in Table 1 of well‐documented large‐scale CF programmes in Bangladesh, Ethiopia, Nepal, Nigeria and Vietnam, and a summary of key findings on related aspects of CF programme design and implementation from a review of implementation research in Table 2. A summary of the lessons learned is provided in the last paragraph of this section.

### 3.1 Large‐Scale CF Programmes in Bangladesh, Ethiopia, Nepal, Nigeria and Vietnam

**Figure 1:**
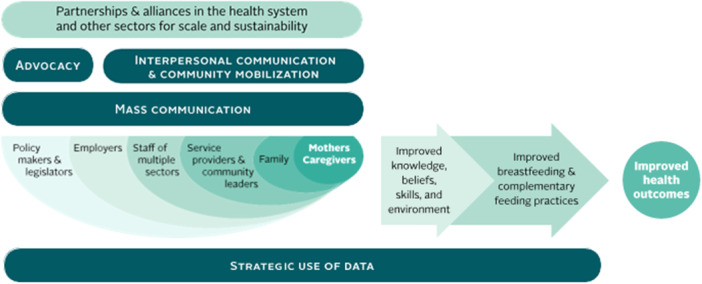
Socioecological model of behaviour change—adapted for infant and young child feeding. Adapted from the socioecological model of behaviour change (Bronfenbrenner 1979), with the addition of operational components and process indicators for designing Alive & Thrive programmes (Baker et al. 2013). *Note:* Mothers and caregivers of children are central within programme strategies; layers of support systems surround them and can enable feeding of infants and young children. The horizontal bars indicate operational programme components needed to effectively engage the various influential levels. The two large arrows on the right connect the mothers and caregivers to outcomes indicating the importance of process indicators (drivers of behaviours) that need to change for achieving improved practices and outcomes. The operational programme components require continuous monitoring and modification based on data as the programmes are implemented and scaled up, to facilitate ongoing improvements in process indicators for achieving improved outcomes.

The country teams designed programmes with a behavioural science lens using the principles of the socioecological model of behaviour change (Figure 1), strengthened nutrition and health care service delivery systems, and implemented them on a large scale. Each programme covered a substantial proportion of populations in need of programme services and implemented them as integral components of existing child health and nutrition programmes and rigorously evaluated them (Flax et al. 2022; Frongillo et al. 2024; Kim et al. 2019; Menon et al. 2016; Rawat et al. 2017). They show that improvements in CF practices are possible in diverse policy, community and programme contexts. Using a behavioural science lens to motivate change and systems strengthening for effective service delivery was common across successful programmes (Baker et al. 2013).

#### 3.1.1 Country Contexts

The Bangladesh, Nepal and Vietnam programmes implemented interventions at or near national scale while the Ethiopia and Nigeria programmes were on a large scale in one region or state per country (Table 1). Scale means deliberate efforts to increase the impact of successfully tested health innovations to benefit more people (WHO 2010b). Government staff implemented the programmes in Ethiopia, Nigeria and Vietnam with technical support from external agencies. In Bangladesh, staff and workers from a large national NGO (BRAC) implemented the programme; in Nepal, staff from government, national and international NGOs implemented the programme, and field staff hired for the project. In Bangladesh, Nigeria and Vietnam, the interventions were embedded in pre‐existing large‐scale community and facility‐based health services. The programmes in Ethiopia and Nepal engaged health services and other sectors: agricultural extension in Ethiopia, agriculture, and water, sanitation, and hygiene in Nepal. The Nepal programme focused on maternal and child nutrition while the others focused on child nutrition. Programme durations ranged from 2 years in Nigeria to 10 years in Nepal. In Nepal, the programme targeted resources to underserviced and disadvantaged communities, whereas the other four programmes served the full population of communities and households in the programme catchment areas, for example, health districts and health centres in Bangladesh, Nigeria and Vietnam and health and agricultural districts in Ethiopia.

The CF practices at the start of the programmes differed widely; minimum dietary diversity scores (MDD) ranged from 5% in Ethiopia to 74% in Vietnam. Knowledge and social norms were the drivers of CF practices across all countries. Nepal and Ethiopia also faced household food insecurity. The programmes engaged influential persons who could address beliefs, food access, and limited time for proper feeding of infants and young children. Formative research was conducted in all countries to prioritize drivers of CF practices, barriers that programmes needed to address, and programme platforms and channels for communication of CF (A&T 2012a, 2012b; Cunningham et al. 2023; Nguyen et al. 2016; Rasheed et al. 2011; Reboot and Picture Impact 2017; Schnefke 2017).

#### 3.1.2 CF Interventions

All countries implemented IPC, community mobilization, and mass media and/or social media activities following systems‐strengthening strategies to reinforce the capacity to deliver programme services and introduced behaviour change interventions to mobilize programme participants, providers and community/family members. Multiple interventions reached specific audiences that could affect CF and reinforce IPC through diverse means to move from knowledge to behaviour change (Hornik 2002). The duration of intervention exposures were 12 months in Nigeria, 2 years in Ethiopia, 4 years in Bangladesh and Vietnam, and 10 years in Nepal,. The content and structure of the interventions were tailored to country resources, needs and practices. IPC involved direct face‐to‐face communication with mothers (counselling) in home visits, community outreach, and/or health facility visits, often through individual dialogue, question‐and‐answer/problem‐solving, hands‐on practice or demonstration, and/or through group sessions. Recognizing the importance of social norms and community‐based supporters of IYCF, each country mobilized existing community networks to build support with family and influential community members. Mass media and social media reached different audiences and large numbers of audiences to generate credibility and emotional appeal, encourage seeking of CF counselling at health centres, increase frequency of exposures through repetition of key messages, and provide reminders. Mass media and social media reached multiple influential groups including front‐line workers and enabled them to provide better quality IPC indicating a synergistic effect of multiple channels (Nguyen, Kim, et al. 2019).

#### 3.1.3 Intervention Coverage and Impact on CF

Coverage of IPC ranged from 11% in Nigeria to over 90% in Bangladesh. Mass media ranged from 25% in Nigeria's Kaduna state to 73% in Bangladesh (Table 1). In Nepal, exposure to mass media had positive associations with dietary diversity for children 6–23.9 and 24–59.9 months in a dose–response manner, and exposure to all three interventions (i.e., interaction with frontline workers, community events and mass media), compared with fewer exposures, had a strong association with child dietary diversity (Suresh et al. 2019). In Bangladesh, **e**xposure to IPC plus mass media was associated with a 1.7–3.5‐fold greater odds of improved CF practices. Exposure to the greatest number of intervention platforms, that is, IPC, mass media, community mobilization events were associated with increased odds of improved CF practices ranging from 2.8 to 5.9‐fold for the different recommended CF practices compared with no exposure (Menon et al. 2016). In Ethiopia, exposure to the number of intervention platforms was associated in a dose–response manner with improved CF knowledge and practices, reduced stunting and improved height for age z‐scores. Exposure to any single intervention was associated with 1.3–2 times higher odds of dietary diversity compared with none; the more platforms to which women were exposed, the higher the odds of dietary diversity among children and exposure to 3 or 4 platforms was associated with 3.2 times higher odds (95% CI: 2.2, 4.6) of dietary diversity (Kim et al. 2019).

### 3.2 Findings of CF Programme Implementation Research Studies on Design and Implementation

#### 3.2.1 Priority CF Practices and Influences

Suboptimal behaviours ranged from too early introduction of foods and liquids in Vietnam, to a combination of early liquids and delayed solids/semi‐solids in other contexts, both leading to low nutrient density in most studies (Table 2). Among determinants, lack of knowledge among health workers and mothers about children's nutrition needs and food quality were almost universal. The studies noted that improving low maternal self‐efficacy and training mothers on careful food selection were top programme priorities across countries. Among health workers, promoting five food groups for dietary diversity overwhelmed mothers and added to low self‐efficacy as compared to picking only 1‐2 missing but accessible individual foods instead of groups. Caregivers identified lack of convenience or time needed to patiently feed infants as they learn to accept CF, particularly when mothers return to work. Influences that mitigated barriers included support by heads of households to procure foods, reminding/reinforcing mothers' new knowledge on CF by family members, and re‐allocating mothers' work to other family members. Purchasing unhealthy, sugary and salty packaged and street foods to please the child or reduce feeding time threatened improving CF. Trials of improved practices, listening to solutions offered by mothers/family members and participatory pilot testing methods were identified as valuable for designing interventions (Dickin et al. 1997; Manoff 2005; PAHO 2013). In Bangladesh and Nigeria, support provided by family members, especially fathers, was important for procuring nutrient‐rich foods, endorsement of new ways of feeding, appropriate use of mothers' time in feeding young children, or sharing mothers' household chores as she fed the child. The role of elder women in the family was also important although male engagement was growing.

#### 3.2.2 Sectors and Programme Platforms Engaged for Delivering CF Interventions

Health services, particularly community‐based volunteers/workers to deliver frequent IPC from 6 to 24 months was the backbone of the programmes. When facility visits were the only option, IPC coverage and impact suffered. Use of platforms such as community‐based organizations and faith‐based organizations in Bangladesh, Ethiopia and Nigeria reached male members and enabled solutions such as setting aside produce from home farms and working around religious fasting through dedicated project components. Bangladesh and Nepal prioritized handwashing and easy access to water at child feeding sites within the home to facilitate adherence and reduce disease transmission from unclean hands and utensils. Ethiopia and Nepal engaged social protection to reach highly vulnerable families. Since mothers often could not make decisions alone, programmes in Bangladesh, Ethiopia, Nepal and Nigeria used non‐health sector structures to engage in family counselling for targeting key decision‐makers such as mothers‐in‐law through women's groups and husbands through trade/profession‐related networks or where men gathered for religious sermons or community dialogue. Social protection programmes were engaged through partnerships with stakeholders in charge of implementation. The breadth of multi‐sectoral decision‐makers, managers and providers plus influential members of the family and community created the need for high‐reach communication channels such as radio, TV, social media through mobile phones, print media and public displays. The programmes contracted commercial advertising agencies to expand coverage and reinforce NGO‐ and government‐led IPC, and community events managed through government, NGO and commercial rural marketing agencies. dose–response analyses of evaluations documented the importance of multiple channels and frequent contacts.

#### 3.2.3 Programme Scale and Costs

Country programmes employed several strategies to reach large scale. First, they integrated CF interventions into existing high‐reach platforms such as primary health care services that already reached communities, enabling community‐based workers to deliver CF interventions, engaging mass media and leveraging community platforms used by family and community influentials. Second, they streamlined and simplified the interventions and tools to encourage adoption by more implementing partners. Third, they aligned the programme with and co‐branded materials with government initiatives and multiple ministries. Fourth, they maintained stakeholder interest by sharing monitoring feedback and human‐interest stories while continuously problem‐solving to mitigate barriers to scale. The costing method used in Nepal differed from that in Bangladesh, Ethiopia and Vietnam. The *Suaahara* project in Nepal calculated costs from a societal perspective and conducted a comprehensive analysis including both financial expenditures incurred by the project and attributed costs (referred to as economic costs); the latter involved assumptions about the value of donated or shared resources, such as time use of existing staff and volunteers and the cost to participants. *Suaahara* included interventions for pregnant women in addition to child nutrition interventions for breastfeeding and CF practices in the first 2 years after birth (Choo et al. 2024). The study calculated costs of activities during the second phase of *Suaahara* (i.e., 2016–2023) and participants reached during 3.7 years of implementation. The average annual total cost was US$908,948 per district, and the programme reached 42 out of the 77 districts in the country. Economic or attributed costs (excluding financial expenditures actually incurred for project interventions) were 47% of total costs and mostly comprised the value of time spent by Female Community Health Workers and participant costs incurred by families to obtain the project services. The proportion of attributed economic costs indicates the extent to which the project successfully utilized existing programme infrastructures and community relationships and strengthened an existing programme. Community events accounted for 29% of the costs and aimed to improve access to and demand for nutritious foods. The programme used home visits to improve maternal and child nutrition practices, accounting for 17% of total costs. Annual economic (attributed) costs plus financial costs (incurred expenditures) ranged from US$132 per participating mother (including pregnant women), to US$76 per mother and child, US$10 per total participant (including all household members) and US$140 per household reached (per year). Personnel (63%) including the time of volunteers were the largest input cost, followed by supplies (11%). If we consider only financial costs incurred by the project for budgeting purposes, the cost would be US$40 per mother and child and US$5 per total participant (including all household members). This programme also improved maternal dietary diversity and water, sanitation and hygiene practices in addition to CF and targeted the hard‐to‐reach communities. The expenditure analysis of Bangladesh and Vietnam programmes covered only CF interventions and only the incremental financial expenditures incurred for strengthening CF interventions and not any costs attributable to pre‐existing infrastructure and did not include interventions for pregnant women. This analysis for the Bangladesh and Vietnam programmes also calculated the cost per child with improved CF practices, in addition to the cost per participant reached. The total annual financial expenditures incurred for the national programmes were US$2,233,931 in Bangladesh and US$958,464 in Vietnam. The expenditure patterns differed in the two countries, with 59% of total expenditures incurred on IPC in Bangladesh as compared to 30% in Vietnam. Mass media accounted for 29% of expenditures in Bangladesh and 30% in Vietnam. The financial expenditures incurred per mother and child reached was US$0.90 in Bangladesh and US$0.93 in Vietnam. The financial expenditures incurred per child with improved CF (minimum acceptable diet) was US$ 6.34 in Bangladesh and US$ 31.39 in Vietnam. External donors financed all expenditures incurred by the five programmes: USAID in Nepal and the Bill & Melinda Gates Foundation in Bangladesh, Ethiopia, Nigeria, and Vietnam.

#### 3.2.4 Factors Affecting Programme Performance, Sustainability and Inequalities

The quality of IPC was dependent on frontline worker attributes such as motivation to act and knowledge. Investing in training, mentoring, supervision, rational workload allocation, streamlined/user‐friendly job aids, monitoring and supervision feedback, incentives and benefits of exposure to other channels such as mass media was valuable in Bangladesh and Vietnam. Experience in Ethiopia showed the vulnerability of cascade training if not diligently followed up to prevent weakening of the training quality (Kim et al. 2015). Identifying existing platforms across sectors with adequate human resources including community health workers (CHWs) to reach families of 6–24‐month‐old infants and children even if they were not highly educated but trainable, and sustaining IPC providers at all levels, particularly CHWs, with frequent contact among peers and programme staff, offering performance incentives, and recognition of satisfactory performance are essential elements. IPC needed to include family contacts and community‐level advocacy to support new CF social norms to obtain the payoffs in the diffusion of improved CF practices through community networks and family support, rather than only depending on health workers for improving and sustaining appropriate CF practices. Although programme activities declined with the removal of external funding, there was evidence of social networks and norms taking over the diffusion function of programmes so that recommended IYCF practices continued 2 years after external funds stopped (Kim, Nguyen, et al. 2018; Kim, Roopnaraine, et al. 2018; Lall et al. 2024). In former intervention areas, dilution of the programme focus due to competing priorities occurred despite sustained increase in provider knowledge gained during the programme period (Moucheraud et al. 2022; Moucheraud et al. 2020). The impact of programmes on socioeconomic inequalities was studied in *Suaahara* in Nepal, and the gaps in MAD between those households with low (i.e., two lowest quintiles) and high (i.e., three highest quintiles) socioeconomic status were reduced in intervention districts by 9.7 pp (*p* = 0.002), whereas no reductions in gaps were seen in comparison districts. In Nepal, aiming to reducing inequalities was stated a priori, and all programme inputs were invested in targeted areas and households (Cunningham et al. 2017; Frongillo et al. 2024). In a study of inequalities in CF practices and programme coverage in endline evaluation surveys in Bangladesh, Ethiopia, Nigeria and Vietnam, inequalities existed both in how children in the 6–23‐months age group were fed and in programmes aimed at improving CF practices, with programmes favoring the better‐off and more educated mothers (Sanghvi et al. 2024). None of these programmes had explicitly aimed to reduce inequalities and did not invest sufficiently in targeting the disadvantaged areas or households unlike the Nepal programme, indicating the importance of taking equity into considerations since programme conception.

### 3.3 Conceptual Model for Large‐Scale CF Programmes

**Figure 2:**
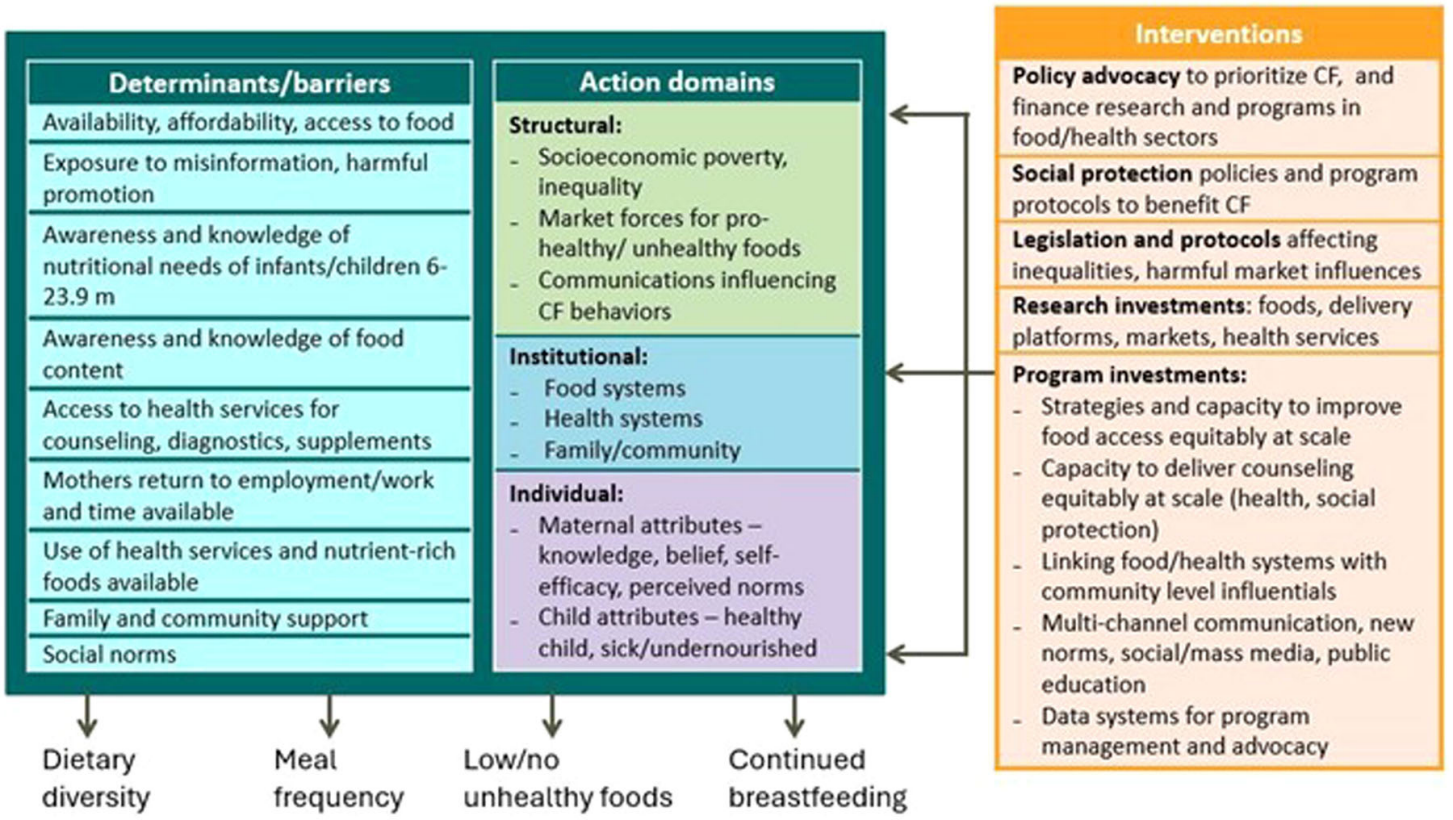
Conceptual framework for strengthening complementary feeding programmes at scale. Adapted from the conceptual model of an enabling environment for breastfeeding (Rollins et al. 2016), the socioecological model of behaviour change (Bronfenbrenner 1979), and UNICEF's Action Framework (UNICEF 2020). *Note:* The relationships illustrated in this figure within and across Determinants/barriers, Action domains and Interventions need to be synchronized, work in cohesion and continually adapt to the environment, for example, shifting norms, food access, mothers' work patterns (Pérez‐Escamilla et al. 2012). CF, complementary feeding; m, months.

The conceptual model illustrates key CF determinants and barriers, action domains or sectors needed to address them, and interventions for operationalization (Figure 2). It is based on common factors documented in implementation research and impact evaluations conducted in the past two decades mostly of the five programmes examined (Table 2). The impact evaluations provided insights on programme attributes associated with intervention coverage and CF outcomes. Implementation research deepened our understanding of critical dimensions such as social norms, family support, food access, maternal self‐efficacy, dose–response impact of multiple channels, sustaining the performance of frontline workers, scale strategies, costs and targeting to reduce inequalities. We drew from conceptual system‐thinking models for breastfeeding protection, promotion and support (Pérez‐Escamilla et al. 2012; Rollins et al. 2016). CF programming needs to emulate the advancement of programme packages for breastfeeding (Pérez‐Escamilla, Dykes, and Kendall 2023; Pérez‐Escamilla, Tomori, et al. 2023). We recommend applying the socioecological model of behaviour change (Figure 1) to map influencing factors at multiple levels in the mothers' environment and specify interventions for each. Each country or location should identify priority CF gaps and underlying determinants combined with feasible operational components appropriate in their own setting before specification of logical frameworks such as theories of change and programme impact pathways of their programmes or embarking on activities to strengthen their existing programmes. A projection of costs and available resources, advocacy for dedicated CF resources, and alliance building to generate needed resources, should occur. A previous breastfeeding programme framework was useful for communicating the comprehensive support needed for mothers to breastfeed optimally (Rollins et al. 2016). The similarity in the programme frameworks for breastfeeding and CF illustrate similar factors needed to enable mothers in feeding infants and young children throughout the first 2 years of life. Just as IPC or counselling alone failed to catalyse breastfeeding improvements, CF requires comprehensive programming. The frameworks for both breastfeeding and CF present multiple dimensions in a single diagram and assume they will work synergistically as highlighted in the breastfeeding gear model which emphasises synchrony and coordination among various action domains and interventions (Pérez‐Escamilla et al. 2012). To facilitate country adaptation of programmes based on Figure 2, we developed a checklist for planning based on lessons learned (Supporting Information Table). The main issues in context‐specific CF programme plans would be determined such as the critical enablers and barriers of CF practices, who key influential persons are besides mothers and caregivers, and which programme platforms offer potential for reaching scale and frequency of contacts/exposures needed for improving and sustaining desirable CF practices.

In Figure 2, the action domains refer to where actions must occur to address determinants and barriers and for delivering services and communications for improving CF practices. The interventions are operational components related to the action domains and involve designing activities based on situational analysis of the needs and opportunities for reaching key participants and motivating them to support CF. Activities may vary according to attributes of the local population, characteristics of food supply, markets and cultural practices, media consumption and communication habits, and strengths and challenges in health services delivery platforms reaching families of the 6–23‐month age group. Social protection, safety net programmes (and emergency programmes including CF in displaced populations) are another means of protecting highly vulnerable children. Research and policy advocacy are critical ongoing overarching elements of effective, self‐correcting, resilient, equitable and sustainable CF programmes.

Key lessons learned for designing and implementing future large scale CF programmes are to use participatory formative studies to develop strategies for engaging selected sectoral stakeholders for specific roles based on coverage gaps and identified barriers; using behavioural science to motivate changes among managers, service providers and families to support mothers in achieving adequate CF practices; and maintaining a results focus through use of data. An understanding of community contexts and existing programme platforms as well as continuous adaptation of programme interventions appeared to facilitate coverage and impacts at scale. Achieving shifts in social norms for CF and systems strengthening to deliver quality interventions through multiple channels showed promising results. Specific targeting of disadvantaged households where child undernutrition is likely to be high was effective in reducing inequalities.

## 4 Discussion

The analysis of programme experiences showed that programme attributes of effective CF programmes were the use of ongoing advocacy to prioritize complementary feeding in key sectors; engagement of stakeholders with high reach, special skills and expertise to address diverse factors such as food access, harmful marketing of unhealthy foods and beverages, knowledge gaps, social norms and maternal self‐efficacy. The lessons learned on implementation of effective CF programmes included continuous monitoring and shaping of interventions for coverage and CF practices, applying a behavioural science lens to address gaps in coverage and CF practices, motivating frontline workers, targeting to reduce inequalities, engaging community leaders and leveraging mass media to reach multiple audiences. The programmes differed across the diverse contexts and included two multi‐sectoral programmes in Ethiopia and Nepal and three health sector‐based programmes in Bangladesh, Nigeria and Vietnam. All achieved improvements in CF. Bangladesh conducted the programme at community level with IPC delivered through home visits and mothers' group education by trained health workers and community volunteers. Programme IPC reached over 90% with multiple visits; mass media reached over 70% of mothers. Vietnam encouraged mothers through mass media messages to visit franchises to receive counselling on CF that were set up within government primary health centres while village health workers visited parents at home and encouraged them to seek counselling at franchises; messages from counselling were more trusted when heard and seen on TV, especially by the grandmothers. Around 40% of eligible mothers ever visited the franchises and among those who did, CF practices improved. Vietnam combined IPC with mass media promotion of iron‐rich foods, recalled by 36% of mothers; media also reached other key influentials. Ethiopia, Nepal and Nigeria used community‐based and outreach by health and in Ethiopia agricultural extension workers also for delivering IPC; they used mass media communication to reinforce IPC and expand coverage. Fathers' engagement in producing (Ethiopia) and procuring nutrient‐rich foods and participating in child feeding was a priority in Bangladesh, Nepal and Nigeria.

We designed the conceptual framework (Figure 2) to facilitate global scale up of effective CF initiatives. Conveying the rationale and conclusions on what works to improve CF is a crucial step. The framework highlights the need for multiple channels and systematically adapted programme components to alleviate CF barriers faced by mothers in each context to streamline and accelerate progress (Pérez‐Escamilla et al. 2012; Pérez‐Escamilla, Dykes, and Kendall 2023; Pérez‐Escamilla, Tomori, et al. 2023; Rollins et al. 2016). To optimize resources, each programme should identify relevant issues, select priorities to enable desirable CF practices, and build upon the pre‐existing intervention elements.

The programmes achieved scale through leveraging the available programme platforms and provided technical and financial resources to strengthen them (Sanghvi et al. 2013). Facility‐based health services tended to be under‐utilized by families once children completed their immunization schedule, and programmes needed additional community‐based service delivery mechanisms. While the programmes successfully raised and sustained knowledge levels of frontline providers, sustaining their motivation to deliver quality services by persevering in detecting and addressing the underlying barriers individual mothers faced needed continued investments. Optimizing household food budgets and resources (as seen in Bangladesh, Nigeria, Vietnam) can cost less than multi‐sectoral initiatives that may require investments in new national and regional platforms for coordination and ongoing training and dialogue with staff from multiple sectors. Field‐level district and subdistrict joint training of health and agricultural extension workers in Ethiopia was effective and focused on a single combined programme theme of using two nutrient‐rich foods from subsistence farms. Similar innovations can be identified by other programme teams through participatory design processes or during scale up through field‐based feedback.

Documenting these lessons learned is timely given a recently issued update on CF recommendations (WHO 2023). The gaps in the diets of young children have also been recently highlighted (UNICEF 2021, 2022). The next step would be to expand the coverage of interventions to support recommended CF practices in different country contexts and reach mothers and families where they are, including at the workplace or on digital platforms to increase the number of contact points to change behaviour. Adopting field‐tested and effective interventions is urgent (Black et al. 2008, 2013). Insights are now available from a group of large‐scale programmes on how best to plan and implement intervention programmes to address a sizeable proportion of the CF problem in different LMICs. Lessons learned indicate the importance of applying behavioural science and nutritional epidemiology in developing content of interventions to improve priority CF practices. Examples are available on how to sequence activities, structure intervention programmes, scale up in phases, and use diverse platforms to reach caregivers and key audiences. The range of impacts on CF practices is now known and we can see what can be expected from programmes in different contexts when underlying factors and the capacity of delivery systems are different. We have documented scaling up strategies for CF programmes. Expenditure analyses have specified the range of budgetary line items needed. What remains to be done is incorporating practical ways to reduce socioeconomic inequalities in intervention coverage as shown in the Nepal programme and promotion of specific foods accessible to families of diverse socioeconomic characteristics. Among limitations, the results of our review aimed at extracting lessons learned were based on an in‐depth understanding of five large‐scale CF models that were documented in detail and, although a literature search provided additional insights from more countries, not all key aspects of design and implementation were reported. Only a handful of rigorous evaluations of impacts on CF practices were available. The consistency in reported experiences across diverse country contexts and programme platforms, however, provides a basis for undertaking more large‐scale programmes in future. The five large programmes in Bangladesh, Ethiopia, Nepal, Nigeria and Vietnam had access to sustained multi‐year financial support and although this vastly improved our understanding of what works, the finding that advocacy was still required for engaging in systems strengthening suggests that advocacy needs to be intensified to prioritize national investments in CF. Further investments in learning are also needed. For example, complex adaptive systems and mixed methods are needed to understand how feedback loops operate at different levels of the socioecological model and explore why the scaling up of some maternal, IYCF programmes are more effective than others (Paina and Peters 2012). This knowledge can speed up cost‐effective adaptation, scaled up implementation, sustainability and dissemination to other settings of evidence‐based CF programmes (Bradley et al. 2012).

## Author Contributions

T.G.S., S.R. and E.A.F. performed the research. T.G.S., S.R. and R.P.‐E. designed the study. C.L., P.P.R., V.O., T.N. and R.M. contributed essential information and data. T.G.S. and E.A.F. wrote the paper with extensive text contributions by C.L. and R.P.‐E. All authors have read and approved the final manuscript.

## Conflicts of Interest

The authors declare no conflicts of interest.

## Supporting information

Supporting information.

## Data Availability

Data sets for coverage data on Bangladesh, Ethiopia, Nigeria and Vietnam from impact evaluations are anonymized and available on A&T's repository on Harvard Dataverse. The data set for Nepal is available from Edward A. Frongillo by writing to EFRONGIL@mailbox.sc.edu. The data that support the findings of this study are openly available in International Food Policy Research Institute (IFPRI) data set at https://dataverse.harvard.edu/dataverse/IFPRI.

## References

[mcn13811-bib-0001] A&T . 2012a. Formative Research on IYCF in Vietnam: Phase I and Phase II Reports. Hanoi.

[mcn13811-bib-0002] A&T . 2012b. Timed and Age‐Appropriate IYCF Messaging for Health Development Army Team Leaders (HDATLs) and Health Extension Workers (HEWs) in Ethiopia. Washington, DC.

[mcn13811-bib-0003] Allotey, D. , V. L. Flax , A. F. Ipadeola , et al. 2022. “Fathers' Complementary Feeding Support Strengthens the Association Between Mothers' Decision‐Making Autonomy and Optimal Complementary Feeding in Nigeria.” Current Developments in Nutrition 6, no. 7: nzac098. 10.1093/cdn/nzac098. 35854939 PMC9283102

[mcn13811-bib-0004] Arimond, M. , and M. T. Ruel . 2004. “Dietary Diversity Is Associated With Child Nutritional Status: Evidence From 11 Demographic and Health Surveys.” Journal of Nutrition 134, no. 10: 2579–2585. 10.1093/jn/134.10.2579. 15465751

[mcn13811-bib-0005] Baker, J. , T. Sanghvi , N. Hajeebhoy , L. Martin , and K. Lapping . 2013. “Using an Evidence‐Based Approach to Design Large‐Scale Programs to Improve Infant and Young Child Feeding.” Food and Nutrition Bulletin 34, no. 3 Suppl: S146–S155. 10.1177/15648265130343s202. 24261073

[mcn13811-bib-0006] Black, M. M. , S. P. Walker , L. C. H. Fernald , et al. 2017. “Early Childhood Development Coming of Age: Science Through the Life Course.” Lancet 389, no. 10064: 77–90. 10.1016/s0140-6736(16)31389-7. 27717614 PMC5884058

[mcn13811-bib-0007] Black, R. E. , L. H. Allen , Z. A. Bhutta , et al. 2008. “Maternal and Child Undernutrition: Global and Regional Exposures and Health Consequences.” Lancet 371, no. 9608: 243–260. 10.1016/s0140-6736(07)61690-0. 18207566

[mcn13811-bib-0008] Black, R. E. , C. G. Victora , S. P. Walker , et al. 2013. “Maternal and Child Undernutrition and Overweight in Low‐Income and Middle‐Income Countries.” Lancet 382, no. 9890: 427–451. 10.1016/s0140-6736(13)60937-x. 23746772

[mcn13811-bib-0009] Boyland, E. , L. McGale , M. Maden , J. Hounsome , A. Boland , and A. Jones . 2022. “Systematic Review of the Effect of Policies to Restrict the Marketing of Foods and Non‐Alcoholic Beverages to Which Children Are Exposed.” Obesity Reviews 23, no. 8: e13447. 10.1111/obr.13447. 35384238 PMC9541016

[mcn13811-bib-0010] Bradley, E. H. , L. A. Curry , L. A. Taylor , et al. 2012. “A Model for Scale up of Family Health Innovations in Low‐Income and Middle‐Income Settings: A Mixed Methods Study.” BMJ Open 2, no. 4: e000987. 10.1136/bmjopen-2012-000987. PMC3432850 22923624

[mcn13811-bib-0011] Bronfenbrenner, U. 1979. The Ecology of Human Development. Cambridge, MA: Harvard University Press.

[mcn13811-bib-0012] Choo, E. M. , C. G. Kemp , K. C. Sagun , et al. 2024. “The Costs of Suaahara II, a Complex Scaled‐Up Multisectoral Nutrition Programme in Nepal.” Maternal & Child Nutrition: e13658. 10.1111/mcn.13658. 38704754 PMC12647970

[mcn13811-bib-0013] Cresswell, J. A. , R. Ganaba , S. Sarrassat , et al. 2017. “Predictors of Exclusive Breastfeeding and Consumption of Soft, Semi‐Solid or Solid Food Among Infants in Boucle du Mouhoun, Burkina Faso: A Cross‐Sectional Survey.” PLoS One 12, no. 6: e0179593. 10.1371/journal.pone.0179593. 28640900 PMC5480894

[mcn13811-bib-0014] Cunningham, K. , D. Nagle , P. Gupta , R. P. Adhikari , and S. Singh . 2021. “Associations Between Parents' Exposure to a Multisectoral Programme and Infant and Young Child Feeding Practices in Nepal.” Maternal & Child Nutrition 17, no. Suppl 1: e13143. 10.1111/mcn.13143. 34241957 PMC8269143

[mcn13811-bib-0015] Cunningham, K. , P. Pandey Rana , M. M. Rahman , A. Sen Gupta , S. Manandhar , and E. A. Frongillo . 2023. “Text Messages to Improve Child Diets: Formative Research Findings and Protocol of a Randomised Controlled Trial in Nepal.” Maternal & Child Nutrition 19, no. 3: e13490. 10.1111/mcn.13490. 36864635 PMC10262875

[mcn13811-bib-0016] Cunningham, K. , A. Singh , P. Pandey Rana , et al. 2017. “Suaahara in Nepal: An At‐Scale, Multi‐Sectoral Nutrition Program Influences Knowledge and Practices While Enhancing Equity.” Maternal & Child Nutrition 13, no. 4: 1–13. 10.1111/mcn.12415. PMC6866152 28058772

[mcn13811-bib-0017] Dickin, K. , M. Griffiths , and E. Piwoz . 1997. Designing by Dialogue: A Program Planners' Guide to Consultative Research for Improving Young ChildFeeding. Washington, DC: https://pdf.usaid.gov/pdf_docs/PNACE296.pdf.

[mcn13811-bib-0018] Dickin, K. L. , K. Litvin , J. K. McCann , and F. M. Coleman . 2021. “Exploring the Influence of Social Norms on Complementary Feeding: A Scoping Review of Observational, Intervention, and Effectiveness Studies.” Current Developments in Nutrition 5, no. 2: nzab001. 10.1093/cdn/nzab001. 33718753 PMC7937492

[mcn13811-bib-0019] Dunford, L. J. , S. C. Langley‐Evans , and S. McMullen . 2012. “Childhood Obesity and Risk of the Adult Metabolic Syndrome: A Systematic Review.” International Journal of Obesity 36, no. 1: 1–11. 10.1038/ijo.2011.186. 22041985 PMC3255098

[mcn13811-bib-0020] Epstein, A. , C. Moucheraud , H. Sarma , et al. 2019. “Does Health Worker Performance Affect Clients' Health Behaviors? A Multilevel Analysis From Bangladesh.” BMC Health Services Research 19, no. 1: 516. 10.1186/s12913-019-4205-z. 31340809 PMC6657138

[mcn13811-bib-0021] Flax, V. L. , A. Ipadeola , C. H. Schnefke , et al. 2022. “Complementary Feeding Social and Behavior Change Communication for Fathers and Mothers Improves Children's Consumption of Fish and Eggs and Minimum Meal Frequency in Kaduna State, Nigeria.” Current Developments in Nutrition 6, no. 5: nzac075. 10.1093/cdn/nzac075. 35669047 PMC9154220

[mcn13811-bib-0022] Frongillo, E. A. 2020. “Designing and Implementing At‐Scale Programs to Improve Complementary Feeding.” Nutrition Reviews 78, no. Suppl 2: 62–70. 10.1093/nutrit/nuz043. 33196087

[mcn13811-bib-0023] Frongillo, E. A. , S. Suresh , D. K. Thapa , et al. 2024. “Impact of Suaahara, an Integrated Nutrition Programme, on Maternal and Child Nutrition at Scale in Nepal.” Maternal & Child Nutrition: e13630. 10.1111/mcn.13630. 38342986 PMC12647975

[mcn13811-bib-0024] Gillespie, S. , P. Menon , and A. L. Kennedy . 2015. “Scaling Up Impact on Nutrition: What Will It Take?” Advances in Nutrition 6, no. 4: 440–451. 10.3945/an.115.008276. 26178028 PMC4496740

[mcn13811-bib-0025] Glenn, J. , C. Moucheraud , D. D. Payán , et al. 2021. “What Is the Impact of Removing Performance‐Based Financial Incentives on Community Health Worker Motivation? A Qualitative Study From an Infant and Young Child Feeding Program in Bangladesh.” BMC Health Services Research 21, no. 1: 979. 10.1186/s12913-021-06996-y. 34535147 PMC8447563

[mcn13811-bib-0026] Hirvonen, K. , and A. Wolle . 2019. *Consumption, Production, Market Access and Affordability of Nutritious Foods in the Afar Region of Ethiopia*. Ethiopia and Washington, DC.

[mcn13811-bib-0027] Hornik, R. C. 2002. Public Health Communication: Evidence for Behavior Change. Lawrence Erlbaum Associates Publishers.

[mcn13811-bib-0028] Kim, S. S. , D. Ali , A. Kennedy , et al. 2015. “Assessing Implementation Fidelity of a Community‐Based Infant and Young Child Feeding Intervention in Ethiopia Identifies Delivery Challenges That Limit Reach to Communities: A Mixed‐Method Process Evaluation Study.” BMC Public Health 15, no. 1: 316. 10.1186/s12889-015-1650-4. 25879417 PMC4392481

[mcn13811-bib-0029] Kim, S. S. , E. M. Mwangi , and R. Rawat. 2015. A&T‐Phase 2 Ethiopia Baseline Report . Washington, DC.

[mcn13811-bib-0030] Kim, S. S. , P. H. Nguyen , L. M. Tran , et al. 2018. “Large‐Scale Social and Behavior Change Communication Interventions Have Sustained Impacts on Infant and Young Child Feeding Knowledge and Practices: Results of a 2‐Year Follow‐Up Study in Bangladesh.” The Journal of Nutrition 148, no. 10: 1605–1614. 10.1093/jn/nxy147. 30169665 PMC6168701

[mcn13811-bib-0031] Kim, S. S. , P. H. Nguyen , L. M. Tran , S. Alayon , P. Menon , and E. A. Frongillo . 2020. “Different Combinations of Behavior Change Interventions and Frequencies of Interpersonal Contacts Are Associated with Infant and Young Child Feeding Practices in Bangladesh, Ethiopia, and Vietnam.” Current Developments in Nutrition 4, no. 2: nzz140. 10.1093/cdn/nzz140. 31976385 PMC6964730

[mcn13811-bib-0032] Kim, S. S. , P. H. Nguyen , Y. Yohannes , et al. 2019. “Behavior Change Interventions Delivered through Interpersonal Communication, Agricultural Activities, Community Mobilization, and Mass Media Increase Complementary Feeding Practices and Reduce Child Stunting in Ethiopia.” Journal of Nutrition 149, no. 8: 1470–1481. 10.1093/jn/nxz087. 31165869 PMC6686053

[mcn13811-bib-0033] Kim, S. S. , R. Rawat , E. M. Mwangi , et al. 2016. “Exposure to Large‐Scale Social and Behavior Change Communication Interventions Is Associated With Improvements in Infant and Young Child Feeding Practices in Ethiopia.” PLoS One 11, no. 10: e0164800. 10.1371/journal.pone.0164800. 27755586 PMC5068829

[mcn13811-bib-0034] Kim, S. S. , T. Roopnaraine , P. H. Nguyen , K. K. Saha , M. I. Bhuiyan , and P. Menon . 2018. “Factors Influencing the Uptake of a Mass Media Intervention to Improve Child Feeding in Bangladesh.” Maternal & Child Nutrition 14, no. 3: e12603. 10.1111/mcn.12603. 29644807 PMC6055868

[mcn13811-bib-0035] Krasevec, J. , X. An , R. Kumapley , F. Bégin , and E. A. Frongillo . 2017. “Diet Quality and Risk of Stunting Among Infants and Young Children in Low‐ and Middle‐Income Countries.” Maternal & Child Nutrition 13, no. S2: 1–11. 10.1111/mcn.12430. PMC6865990 29032628

[mcn13811-bib-0036] Lall, G. , E. A. Frongillo , P. H. Nguyen , S. S. Kim , and P. Menon . 2024. “Changes in Norms About Infant and Young Child Feeding in Rural Bangladesh During the Alive & Thrive Initiative.” Current Developments in Nutrition 8: 102961. 10.1016/j.cdnut.2024.102961.

[mcn13811-bib-0037] Laving, A. R. , S. R. Hussain , and D. O. Atieno . 2018. “Overnutrition: Does Complementary Feeding Play a Role?” Annals of Nutrition and Metabolism 73, no. Suppl 1: 15–18. 10.1159/000490088. 30196296

[mcn13811-bib-0038] Lutter, C. K. , L. Grummer‐Strawn , and L. Rogers . 2021. “Complementary Feeding of Infants and Young Children 6 to 23 Months of Age.” Nutrition Reviews 79, no. 8: 825–846. 10.1093/nutrit/nuaa143. 33684940

[mcn13811-bib-0039] Manoff . 2005. Trials of Improved Practices (TIPS) . www.behaviourchange.net.

[mcn13811-bib-0040] Menon, P. , P. H. Nguyen , K. K. Saha , et al. 2016. “Combining Intensive Counseling by Frontline Workers With a Nationwide Mass Media Campaign Has Large Differential Impacts on Complementary Feeding Practices But Not on Child Growth: Results of a Cluster‐Randomized Program Evaluation in Bangladesh.” Journal of Nutrition 146, no. 10: 2075–2084. 10.3945/jn.116.232314. 27581575 PMC5037872

[mcn13811-bib-0041] Moucheraud, C. , A. Epstein , H. Sarma , et al. 2022. “Assessing Sustainment of Health Worker Outcomes Beyond Program End: Evaluation Results From an Infant and Young Child Feeding Intervention in Bangladesh.” Frontiers in Health Services 2: 1005986. 10.3389/frhs.2022.1005986. 36925817 PMC10012630

[mcn13811-bib-0042] Moucheraud, C. , H. Sarma , T. T. T. Ha , et al. 2020. “Can Complex Programs be Sustained? A Mixed Methods Sustainability Evaluation of a National Infant and Young Child Feeding Program in Bangladesh and Vietnam.” BMC Public Health 20, no. 1: 1361. 10.1186/s12889-020-09438-2. 32887601 PMC7487916

[mcn13811-bib-0043] Nguyen, P. H. , E. A. Frongillo , S. S. Kim , et al. 2019. “Information Diffusion and Social Norms Are Associated With Infant and Young Child Feeding Practices in Bangladesh.” Journal of Nutrition 149, no. 11: 2034–2045. 10.1093/jn/nxz167. 31396621 PMC6825823

[mcn13811-bib-0044] Nguyen, P. H. , S. S. Kim , T. T. Nguyen , et al. 2016. “Supply‐ and Demand‐Side Factors Influencing Utilization of Infant and Young Child Feeding Counselling Services in Viet Nam.” PLoS One 11, no. 3: e0151358. 10.1371/journal.pone.0151358. 26962856 PMC4786102

[mcn13811-bib-0045] Nguyen, P. H. , S. S. Kim , L. M. Tran , P. Menon , and E. A. Frongillo . 2019. “Intervention Design Elements Are Associated With Frontline Health Workers' Performance to Deliver Infant and Young Child Nutrition Services in Bangladesh and Vietnam.” Current Developments in Nutrition 3, no. 8: 3008001. 10.1093/cdn/nzz070. PMC6642067 31346584

[mcn13811-bib-0046] Nguyen, P. H. , S. Manohar , L. Mai , A. Subandoro , R. Rawat , and P. Menon . 2011. Alive & Thrive Baseline Survey Report . Viet Nam, Washington, DC.

[mcn13811-bib-0047] Nguyen, P. H. , P. Menon , S. C. Keithly , et al. 2014. “Program Impact Pathway Analysis of a Social Franchise Model Shows Potential to Improve Infant and Young Child Feeding Practices in Vietnam.” Journal of Nutrition 144, no. 10: 1627–1636. 10.3945/jn.114.194464. 25143372

[mcn13811-bib-0048] Nguyen, P. H. , P. Menon , M. Ruel , and N. Hajeebhoy . 2011. “A Situational Review of Infant and Young Child Feeding Practices and Interventions in Viet Nam.” Asia Pacific Journal of Clinical Nutrition 20, no. 3: 359–374. 21859654

[mcn13811-bib-0049] Nguyen, T. T. , N. Hajeebhoy , J. Li , C. T. Do , R. Mathisen , and E. A. Frongillo . 2021. “Community Support Model on Breastfeeding and Complementary Feeding Practices in Remote Areas in Vietnam: Implementation, Cost, and Effectiveness.” International Journal for Equity in Health 20, no. 1: 121. 10.1186/s12939-021-01451-0. 34001154 PMC8127246

[mcn13811-bib-0050] PAHO . 2013. ProPAN: Process for the Promotion of Child Feeding . Washington, DC. https://www.paho.org/en/documents/propan-process-promotion-child-feeding-field-manual.

[mcn13811-bib-0051] Paina, L. , and D. H. Peters . 2012. “Understanding Pathways for Scaling Up Health Services Through the Lens of Complex Adaptive Systems.” Health Policy and Planning 27, no. 5: 365–373. 10.1093/heapol/czr054. 21821667

[mcn13811-bib-0052] Pérez‐Escamilla, R. , O. Bermudez , G. S. Buccini , et al. 2018. “Nutrition Disparities and the Global Burden of Malnutrition.” BMJ 361: k2252. 10.1136/bmj.k2252. 29899012 PMC5996967

[mcn13811-bib-0053] Pérez‐Escamilla, R. , L. Curry , D. Minhas , L. Taylor , and E. Bradley . 2012. “Scaling Up of Breastfeeding Promotion Programs in Low‐ and Middle‐Income Countries: The ‘Breastfeeding Gear’ Model.” Advances in Nutrition 3, no. 6: 790–800. 10.3945/an.112.002873. 23153733 PMC3648703

[mcn13811-bib-0054] Pérez‐Escamilla, R. , F. C. Dykes , and S. Kendall . 2023. “Gearing to Success With National Breastfeeding Programmes: The Becoming Breastfeeding Friendly (BBF) Initiative Experience.” Maternal & Child Nutrition 19, no. Suppl 1: e13339. 10.1111/mcn.13339. 35254735 PMC9835584

[mcn13811-bib-0055] Pérez‐Escamilla, R. , E. Y. Jimenez , and K. G. Dewey . 2021. “Responsive Feeding Recommendations: Harmonizing Integration Into Dietary Guidelines for Infants and Young Children.” Current Developments in Nutrition 5, no. 6: nzab076. 10.1093/cdn/nzab076. 34104850 PMC8178105

[mcn13811-bib-0056] Pérez‐Escamilla, R. , C. Tomori , S. Hernández‐Cordero , et al. 2023. “Breastfeeding: Crucially Important, But Increasingly Challenged in a Market‐Driven World.” Lancet 401, no. 10375: 472–485. 10.1016/S0140-6736(22)01932-8. 36764313

[mcn13811-bib-0057] Rasheed, S. , R. Haider , N. Hassan , et al. 2011. “Why Does Nutrition Deteriorate Rapidly Among Children Under 2 Years of Age? Using Qualitative Methods to Understand Community Perspectives on Complementary Feeding Practices in Bangladesh.” Food and Nutrition Bulletin 32, no. 3: 192–200. 10.1177/156482651103200302. 22073792

[mcn13811-bib-0058] Rasmi, A. , M. Purnima , S. Kuntal K., et al. 2013. “A Program Impact Pathway Analysis Identifies Critical Steps in the Implementation and Utilization of a Behavior Change Communication Intervention Promoting Infant and Child Feeding Practices in Bangladesh.” Journal of Nutrition 143, no. 12: 2029–2037. 10.3945/jn.113.179085. 24068790

[mcn13811-bib-0059] Rawat, R. , P. H. Nguyen , L. M. Tran , et al. 2017. “Social Franchising and a Nationwide Mass Media Campaign Increased the Prevalence of Adequate Complementary Feeding in Vietnam: A Cluster‐Randomized Program Evaluation.” Journal of Nutrition 147, no. 4: 670–679. 10.3945/jn.116.243907. 28179488 PMC5368587

[mcn13811-bib-0060] Reboot and Picture Impact . 2017. A&T Nigeria Formative Research Findings Report . FHI 360, Washington DC.

[mcn13811-bib-0061] Rollins, N. , E. Piwoz , P. Baker , et al. 2023. “Marketing of Commercial Milk Formula: A System to Capture Parents, Communities, Science, and Policy.” Lancet 401, no. 10375: 486–502. 10.1016/s0140-6736(22)01931-6. 36764314

[mcn13811-bib-0062] Rollins, N. C. , N. Bhandari , N. Hajeebhoy , et al. 2016. “Why Invest, and What It Will Take to Improve Breastfeeding Practices?” Lancet 387, no. 10017: 491–504. 10.1016/S0140-6736(15)01044-2. 26869576

[mcn13811-bib-0063] Sanghvi, T. , R. Haque , S. Roy , et al. 2016. “Achieving Behaviour Change at Scale: Alive & Thrive's Infant and Young Child Feeding Programme in Bangladesh.” Maternal & Child Nutrition 12, no. Suppl 1: 141–154. 10.1111/mcn.12277. 27187912 PMC6680185

[mcn13811-bib-0064] Sanghvi, T. , L. Martin , N. Hajeebhoy , et al. 2013. “Strengthening Systems to Support Mothers in Infant and Young Child Feeding at Scale.” Food and Nutrition Bulletin 34, no. 3 Suppl 2: S156–S168. 10.1177/15648265130343s203. 24261074

[mcn13811-bib-0065] Sanghvi, T. , R. Seidel , J. Baker , and A. Jimerson . 2017. “Using Behavior Change Approaches to Improve Complementary Feeding Practices.” Maternal & Child Nutrition 13, no. Suppl 2: 1–11. 10.1111/mcn.12406. PMC6865988 29032626

[mcn13811-bib-0066] Sanghvi, T. G. , D. Godha , and E. A. Frongillo . 2024. “Inequalities in Complementary Feeding Programs in Randomized Intervention and Nonintervention Areas After Program Implementation in Bangladesh, Ethiopia, and Vietnam.” Current Developments in Nutrition 8, no. 9: 104426. 10.1016/j.cdnut.2024.104426. 39263223 PMC11388651

[mcn13811-bib-0067] Savin, M. , A. Vrkatić , D. Dedić , et al. 2022. “Additives in Children's Nutrition—A Review of Current Events.” International Journal of Environmental Research and Public Health 19, no. 20: 13452. 10.3390/ijerph192013452. 36294032 PMC9603407

[mcn13811-bib-0068] Schnefke , C. H. , V. L. Flax , O. Daniel , and C. K. Bowman . 2017. Alive & Thrive Nigeria: Baseline Qualitative Data Report (Kaduna and Lagos) . Washington, DC.

[mcn13811-bib-0069] Schneider, B. C. , G. Gatica‐Domínguez , M. C. F. Assunção , et al. 2020. “Introduction to Complementary Feeding in the First Year of Life and Risk of Overweight at 24 Months of Age: Changes From 2004 to 2015 Pelotas (Brazil) Birth Cohorts.” British Journal of Nutrition 124, no. 6: 620–630. 10.1017/s0007114520001634. 32381141

[mcn13811-bib-0070] Smith, R. , B. Kelly , H. Yeatman , and E. Boyland . 2019. “Food Marketing Influences Children's Attitudes, Preferences and Consumption: A Systematic Critical Review.” Nutrients 11, no. 4: 875. 10.3390/nu11040875. 31003489 PMC6520952

[mcn13811-bib-0071] Suresh, S. , A. Paxton , B. K. Pun , et al. 2019. “Degree of Exposure to Interventions Influences Maternal and Child Dietary Practices: Evidence From a Large‐Scale Multisectoral Nutrition Program.” PLoS One 14, no. 8: e0221260. 10.1371/journal.pone.0221260. 31449529 PMC6709950

[mcn13811-bib-0072] Thapa, D. K. , E. A. Frongillo , S. Suresh , et al. 2024. “Impact of *Suaahara*, an At‐Scale Multisectoral Nutrition Programme, on Health Workers' Maternal and Child Health, and Nutrition Knowledge and Skills in Nepal.” Maternal & Child Nutrition: e13669. 10.1111/mcn.13669. 38881273 PMC12647983

[mcn13811-bib-0073] UNICEF . 2020. *Improving Young Children's Diets During the Complementary Feeding Period*. UNICEF Programming Guidance. New York, NY.

[mcn13811-bib-0074] UNICEF . 2021. *Fed to Fail? The Crisis of Children's Diets in Early Life*. Child Nutrition Report. UNICEF.

[mcn13811-bib-0075] UNICEF . 2022. Child Food Poverty: A Nutrition Crisis in Early Childhood . New York, NY.

[mcn13811-bib-0076] Victora, C. G. , M. de Onis , P. C. Hallal , M. Blössner , and R. Shrimpton . 2010. “Worldwide Timing of Growth Faltering: Revisiting Implications for Interventions.” Pediatrics 125, no. 3: e473–e480. 10.1542/peds.2009-1519. 20156903

[mcn13811-bib-0077] Warren, A. M. , E. A. Frongillo , P. H. Nguyen , and P. Menon . 2020. “Nutrition Intervention Using Behavioral Change Communication Without Additional Material Inputs Increased Expenditures on Key Food Groups in Bangladesh.” Journal of Nutrition 150, no. 5: 1284–1290. 10.1093/jn/nxz339. 31943055 PMC7198287

[mcn13811-bib-0078] WHO . 2010a. Indicators for Assessing Infant and Young Child Feeding Practices: Part 2: Measurement . Geneva, Switzerland.

[mcn13811-bib-0079] WHO . 2010b. Nine Steps for Developing a Scaling‐Up Strategy. World Health Organization.

[mcn13811-bib-0080] WHO . 2023. WHO Guideline for Complementary Feeding of Infants and Young Children 6–23 Months of Age . Geneva, Switzerland. 37871145

[mcn13811-bib-0081] WHO, UNICEF, FAO, WFP, & UNHCR . 2020. Global Action Plan on Child Wasting: A Framework for Action to Accelerate Progress in Preventing and Managing Child Wasting and The Achievement of the Sustainable Development Goals . https://www.who.int/publications/m/item/global-action-plan-on-child-wasting-a-framework-for-action.

