# Supplementary material for: Evidence‐Based Lessons From Two Decades of Implementation Research on Complementary Feeding Programmes
Source: Matern Child Nutr. 2025 Feb 28;21(3):e13811. doi: 10.1111/mcn.13811 (PMC12150138; doi:10.1111/mcn.13811)
Supplement: Supplementary file 1 — Supporting information. [file MCN-21-e13811-s001.docx]

**SUPPLEMENTARY MATERIAL: APPENDIX**

**Checklist for Documenting Country and Program Level Complementary Feeding Practices, Determinants and Program Platforms to Plan Contextualized Programs**

This checklist provides a quick way to document how program content and implementation arrangements need to be adapted for specific program locations. It aims to facilitate the planning of locally relevant program content and activities. It is organized around the conceptual framework in the manuscript (Figure 2). The checklist can be used by decision-makers, program developers, or managers responsible for planning or implementing a CF project and who wish to identify the most important factors for achieving results in their setting.

**How the checklist works**

A plus (+) refers to an important success factor that should be leveraged for achieving results at scale. A minus (–) refers to a barrier that needs to be mitigated or removed. Depending upon whether the issue is a facilitating factor or an obstruction or challenge, answer each question by placing a check in the plus or minus column. In both cases, the program needs to consider interventions and/or implementing arrangements to address the factor. The relative importance of issues can be indicated by using multiple checks in plus or minus columns up to a maximum of three. Consider the quality of evidence used to make these judgements when filling the checklist including representativeness and how recent the existing information/studies are. If satisfactory information is not available on a question, the project team may want to note how the information gap can be filled.

**Country Adaptation of Complementary Feeding Programs: Questions for Setting Priorities**

| **Questions About CF Practices and Determinants** | **Enablers (+)** | **Barriers (–)** | **Data sources/gaps** |
| --- | --- | --- | --- |
| **Availability, affordability, access to food** |  |  |  |
| - Is household food security adequate for CF? |  |  |  |
| - Are there foods in local markets to meet dietary diversity recommendations? |  |  |  |
| - Is only one food group missing or more? |  |  |  |
| - Are seasonal availability and/or prices of recommended foods stable? |  |  |  |
| **Access to health services for counseling, diagnostics, supplements** |  |  |  |
| - Is geographic distance to primary health care facilities a reason for missing CF counseling? |  |  |  |
| - Are health outreach services for CF counselling available in the communities from any source? |  |  |  |
| - Are mothers of young children permitted to participate freely in education/counseling at health facilities and community events? |  |  |  |
| **Awareness and knowledge of nutritional needs, timing, diversity, type, amounts, responsive feeding** |  |  |  |
| - Among mothers? |  |  |  |
| - Among fathers and family members? |  |  |  |
| - Among health workers? |  |  |  |
| - Among program authorities? |  |  |  |
| **Awareness and knowledge of food content** |  |  |  |
| - Are mothers selecting appropriate foods? |  |  |  |
| - Are family members procuring healthy foods in adequate amounts to meet children’s needs? |  |  |  |
| **Exposure to accurate/contradictory information** |  |  |  |
| - Do mass media and/or social media provide accurate information on CF? |  |  |  |
| - Do food labels and product promotions in markets or shops provide useful information to guide healthy CF choices? |  |  |  |
| - Are there regulations to protect families from product misinformation and false health claims? |  |  |  |
| - Do health providers advise/counsel/motivate caregivers on healthy foods? |  |  |  |
| - Are health providers held accountable for misinformation or false promotion of products? |  |  |  |
| - Do mothers know where and how to obtain reliable information on CF? |  |  |  |
| - How many channels/sources can be used to deliver reliable messages to mothers? |  |  |  |
| **Mothers employment/work and time available** |  |  |  |
| - To alleviate workload for mothers are other caregivers available/engaged in CF activities? |  |  |  |
| - Are unhealthy convenience foods considered adequate and used in CF to save time? |  |  |  |
| - Are mothers and/or the usual caregivers skilled and confident in managing healthy CF options that are convenient? |  |  |  |
| **Family and community support** |  |  |  |
| - Are fathers/male members engaged and informed about CF needs and provide tangible support? |  |  |  |
| - Are other influential members of the family and community informed about CF needs and provide tangible support |  |  |  |
| - Do mothers perceive and report that family and community members are supportive of recommended CF practices? |  |  |  |
| **Social norms** |  |  |  |
| - Are timely introduction of CF, diversity, meal frequency and solid/semi-solid foods normative practices in the communities? |  |  |  |
| - Do mothers perceive/believe and report that the recommended CF practices do not conflict with social norms? |  |  |  |
| - Are mothers confident about resolving differences between recommended and family/community norms? |  |  |  |
| **Questions About Action Domains** | **Enablers (+)** | **Barriers (–)** | **Data sources/gaps** |
| **Structural/policy level:** |  |  |  |
| -Are there well-defined and widely known policies for enabling reductions in socioeconomic inequalities that affect CF practices? |  |  |  |
| -Are there policies to enable market forces that are supportive of affordable and accessible healthy foods for CF? |  |  |  |
| -Are there policies to control market forces that encourage unhealthy foods for CF? |  |  |  |
| -Does the government facilitate communications on healthy CF practices to reach urban and rural families of young children of all socioeconomic groups? |  |  |  |
| **Institutional level:** |  |  |  |
| - *Food systems* |  |  |  |
| Have food-based strategies been considered for addressing CF gaps in selected geographic areas and/or socioeconomic groups? |  |  |  |
| For strategies involving food supply and distribution to improve CF, are human/financial resources and management adequate to reach/maintain targets? |  |  |  |
| What percentage of mothers can be reached through food systems (e.g. markets, ag. extension, farm schools) |  |  |  |
| - *Health systems* |  |  |  |
| Do routine health primary health services include regular contact with families of children in the 6-23.9-month age group, and is counseling on CF adequately integrated and given priority? |  |  |  |
| What percent of mothers of 6-23.9 months old children can be reached through health services? |  |  |  |
| Are human/financial resources and management adequate to reach/maintain targets for providing counseling, screening and supplementation of children in the 6-23.9-month age group? |  |  |  |
| - Do primary health care services prioritize counseling or care for children with poor diets or growth deficits? |  |  |  |
| - Do primary health care services provide a balanced approach to prevention of wasting through CF and treatment of wasting? |  |  |  |
| - *Family/community* |  |  |  |
| What networks and platforms are available for reaching fathers of 6-23.9 months old children? |  |  |  |
| What community networks and platforms can reach key influentials who can support CF? |  |  |  |
| **Individual mothers’ level:** |  |  |  |
| - Are mothers’ knowledge of children’s CF needs and recommended CF practices a major gap? |  |  |  |
| - Is belief in the benefits of CF or self-efficacy/confidence to practice the CF or perception of social norms by mothers the main gaps? |  |  |  |
| - Do mothers know how to assess and address ‘poor appetite’? |  |  |  |
| - Do mothers understand that children can consume the recommended foods in adequate amounts? |  |  |  |
| - Do mothers know that excessively sweet or salty foods can predispose children to unhealthy foods? |  |  |  |
| **Questions About Interventions** | **Enablers (+)** | **Barriers (–)** | **Data sources/gaps** |
| **Policy advocacy:** |  |  |  |
| -Are there policy advocacy initiatives to prioritize CF as a development issue and establish targets/goals to support CF through relevant sectors? |  |  |  |
| -Is there effective policy advocacy to invest adequate financial resources in the food, health, and social protection sectors dedicated to improving CF? |  |  |  |
| -Is there legislation and policy initiatives for reducing inequalities affecting nutrition of 6-23.9-month-old children? |  |  |  |
| -Is there legislation and policy initiatives for reducing harmful market influences? |  |  |  |
| **Research investments:** |  |  |  |
| -Are investments needed in and adequate for research on nutrient-rich foods and food preferences? |  |  |  |
| - Are investments needed in and adequate for research on access to and markets for CF foods? |  |  |  |
| - Are investments needed in and adequate for research on health services delivery platforms for 6-23.9-month-old children? |  |  |  |
| **Capacity to deliver programs:** |  |  |  |
| -Are there adequate systems-strengthening strategies and capacity to improve food access for CF equitably at scale? |  |  |  |
| - Is there adequate capacity to deliver CF counseling equitably at scale through health and social protection programs? |  |  |  |
| -Are there adequate linkages between food/health systems and community level influentials for engagement in CF programs? |  |  |  |
| -Is there adequate use of multi-channel communication and social/mass media for ongoing public education on CF? |  |  |  |
| -Are there adequate data systems for accountability, management and advocacy for CF programs? |  |  |  |

*Abbreviation: CF=Complementary feeding*
